# Secretagogin as a marker to distinguish between different neuron types in human frontal and temporal cortex

**DOI:** 10.3389/fnana.2023.1210502

**Published:** 2023-11-01

**Authors:** Silvia Tapia-González, Javier DeFelipe

**Affiliations:** ^1^Laboratorio Cajal de Circuitos Corticales, Centro de Tecnología Biomédica, Universidad Politécnica de Madrid, Madrid, Spain; ^2^Instituto Cajal, Consejo Superior de Investigaciones Científicas (CSIC), Madrid, Spain; ^3^Laboratorio de Neurofisiología Celular, Facultad de Medicina, Universidad San Pablo-CEU, CEU Universities, Madrid, Spain; ^4^Centro de Investigación Biomédica en Red sobre Enfermedades Neurodegenerativas (CIBERNED), ISCIII, Madrid, Spain

**Keywords:** calcium binding proteins, colocalization, triple immunostaining, parvalbumin, calretinin, nitric oxide synthase, interneurons, cortical layers

## Abstract

The principal aim of the present work was to chemically characterize the population of neurons labeled for the calcium binding protein secretagogin (SCGN) in the human frontal and temporal cortices (Brodmann’s area 10 and 21, respectively). Both cortical regions are involved in many high cognitive functions that are especially well developed (or unique) in humans, but with different functional roles. The pattern of SCGN immunostaining was rather similar in BA10 and BA21, with all the labeled neurons displaying a non-pyramidal morphology (interneurons). Although SCGN cells were present throughout all layers, they were more frequently observed in layers II, III and IV, whereas in layer I they were found only occasionally. We examined the degree of colocalization of SCGN with parvalbumin (PV) and calretinin (CR), as well as with nitric oxide synthase (nNOS; the enzyme responsible for the synthesis of nitric oxide by neurons) by triple immunostaining. We looked for possible similarities or differences in the coexpression patterns of SCGN with PV, CR and nNOS between BA10 and BA21 throughout the different cortical layers (I–VI). The percentage of colocalization was estimated by counting the number of all labeled cells through columns (1,100–1,400 μm wide) across the entire thickness of the cortex (from the pial surface to the white matter) in 50 μm-thick sections. Several hundred neurons were examined in both cortical regions. We found that SCGN cells include multiple neurochemical subtypes, whose abundance varies according to the cortical area and layer. The present results further highlight the regional specialization of cortical neurons and underline the importance of performing additional experiments to characterize the subpopulation of SCGN cells in the human cerebral cortex in greater detail.

## 1. Introduction

Calcium (Ca2+) is involved in many critical functions of cellular physiology and the biochemistry of organisms. In the nervous system, Ca2+ plays a crucial role in a wide range of functions, including neuronal plasticity and synaptic function. As discussed in Alpar et al. (2012), several mechanisms are involved in the homeostatic control of Ca2+, including those mediated by Ca2 + −binding proteins (CBPs). The discovery and localization of CBPs in the nervous system during the 1980s represented an important step in the study of various aspects of the functional and structural organization of brain circuits. A variety of CBPs have been shown to be present in particular subpopulations of neurons in multiple functional systems of the vertebrate nervous system (Celio, 1990; Baimbridge et al., 1992; Jones, 1998; Alpar et al., 2012; Gati et al., 2014). In the cerebral cortex, the CBPs parvalbumin (PV), calbindin-D28k (CalB) and calretinin (CR) represent the most commonly used markers to discern between different morphological, neurochemical and physiological types of cortical neurons in numerous mammalian species, including humans (reviewed in Andressen et al., 1993; DeFelipe, 1993; Freund and Buzsaki, 1996; DeFelipe, 1997; Hof et al., 1999; Markram et al., 2004; Ascoli et al., 2008; Burkhalter, 2008; Caputi et al., 2013; Kubota, 2014; Staiger et al., 2015; Tremblay et al., 2016; Boldog et al., 2018; Hodge et al., 2019; Yuste et al., 2020). In general, these CBPs are expressed (for simplicity, +) in different populations of GABAergic interneurons, but certain CalB+ and CR+ neurons do not express (for simplicity, −) GABA, depending on the cortical region and species. For example, a large subpopulation of pyramidal neurons is CalB+ in CA1 of mouse, but not in human (see Merino-Serrais et al., 2020). In the neocortex of rodents, almost 100% of CR+ neurons have been reported to be also immunostained for GABA (Kubota et al., 1994; Gonchar and Burkhalter, 1997; Gonchar et al., 2007; Kubota et al., 2011), but in the neocortex of monkey (Melchitzky et al., 1999) and human (del Rio and DeFelipe, 1996), approximately 25% of CR+ neurons do not express GABA.

Secretagogin (SCGN) is another CBP that was discovered more recently (Archer and Wagner, 2000; Wagner et al., 2000). The use of this CBP is becoming increasingly popular in neurobiology because: (1) it may help to further investigate new aspects of Ca2+ signaling in both normal and altered nervous system and (2) it is expressed in different types of cells in multiple regions of the adult and the developing nervous system of a variety of species (Gartner et al., 2001; Alpar et al., 2012; Gati et al., 2014; Raju et al., 2018; Maj et al., 2019; Alzu'bi and Clowry, 2020; Qin et al., 2020).

From the neuroanatomical and functional point of view, this second characteristic is important as it may facilitate the discovery of similarities and differences between particular subsets of neurons in different regions of the nervous system of a particular species or between different species. For example, Garas et al. (2016) have reported that the co-expression of SCGN and PV can be used to subdivide the population of PV+ interneurons into two topographically-, physiologically- and structurally-distinct cell populations in the striatum of both rats and primates. These two subpopulations also differ with regard to their connections: the cell body of spiny projection neurons of the direct and indirect pathway are preferentially innervated by PV+/SCGN+ and PV+/SCGN− axons, respectively. In another study, Kosaka et al. (2017) reported that 60–70% of SCGN+ neurons in the rat striatum are either PV+, CR+ or ChAT+ (choline acetyl transferase), but none contained nitric oxide synthase (nNOS). Thus, these authors concluded that these populations of interneurons could play different roles in the inhibition of striatal outputs and are therefore likely to be involved in different aspects of the control of behavior.

Recently, we have examined the pattern of SCGN-immunoreactivity in different hippocampal fields (DG, CA1, CA2, CA3, subiculum, presubiculum, and parasubiculum) as well as in the entorhinal and perirhinal cortex of human, rat, and mouse (Tapia-Gonzalez et al., 2020). We found significant similarities and differences in the pattern of labeling among the human, rat, and mouse in these brain regions, as well as between other brain regions examined within each species. Such regional variations might reflect important differences in circuit structure contributing to regional differences in cortical function. The principal aim of the present work was to morphologically and chemically characterize the population of SCGN+ cells in other cortical regions of the human brain for which there is no information – or if information does exist, it is very limited. In particular, we examined the degree of colocalization of SCGN with PV and CR as well as with nNOS by triple immunostaining in two different cortical regions: Brodmann’s area 10 (BA10; also known as the frontal pole or rostral/anterior prefrontal cortex), and Brodmann’s area 21 (BA21; approximately correspond to the anterior middle temporal gyrus; see Zilles and Amunts, 2010). Both cortical regions are involved in many high cognitive functions that are especially well developed (or unique) in humans, but with different functional roles; for example, planning of future actions in the prefrontal cortex or semantic processing, language, and theory of mind in the temporal cortex (Damasio et al., 1996; Semendeferi et al., 2002; Kaas, 2012, 2013; Braunsdorf et al., 2021; Fuster, 2021; Friedman and Robbins, 2022; Preuss and Wise, 2022). Therefore, we were looking for possible similarities or differences in the coexpression patterns of SCGN with PV, CR and nNOS between BA10 and BA21 throughout the different cortical layers (I–VI). We found that SCGN+ cells include multiple neurochemical subtypes whose abundance varies according to the cortical area and layer.

## 2. Materials and methods

### 2.1. Tissue preparation

Human brain tissue was obtained at autopsy from the Unidad Asociada Neuromax (Laboratorio de Neuroanatomía Humana, Facultad de Medicina, Universidad de Castilla-La Mancha, Albacete, and the Laboratorio Cajal de Circuitos Corticales UPM-CSIC, Madrid, Spain). The tissue was obtained following national laws and international ethical and technical guidelines on the use of human samples for biomedical research purposes. In this study, we used samples of human brain tissue from 4 control human brains (subjects with no recorded neurological or psychiatric alterations): 3 males, aged 45 (AB1), 53 (AB3), and 66 (AB7) years old, and 1 female, aged 53 years old (AB2). The post-mortem time (PT) between death and brain fixation varied between 1.5 and 4 h (AB1: 1.5 h; AB3: 3.5 h; AB2: 4 h; and AB7: 4 h PT). Tissue from human brains AB1, AB2, and AB3 has been used in previous studies (Anton-Fernandez et al., 2017; Benavides-Piccione et al., 2020; Tapia-Gonzalez et al., 2020). The cause of death was pleural mesothelioma (case AB1), septic shock of pulmonary origin (case AB2), and metastatic bladder carcinoma (cases AB3 and AB7). Upon removal, the brains (AB2, AB3, and AB7) were immediately fixed in cold 4% paraformaldehyde (PFA) in 0.1 mol/L, pH 7.4 phosphate buffer (PB), and sectioned into 1.5 cm-thick coronal slabs. The prefrontal (BA10) and temporal (BA21) cortical areas were cut into approximately 1 cm × 1 cm × 1 cm blocks and post-fixed in the same fixative for 24–48 h at 4°C. Case AB1 was perfused through both internal carotid arteries <1 h after death with a saline solution followed by 4% PFA in PB. The brain was then removed and post-fixed as described above. The tissue blocks were subsequently cryoprotected in 25% sucrose in PB and stored at −20°C in a solution of glycerol, ethylene glycol and PB. Serial neocortical sections (50 μm) of each case were obtained using a vibratome (Leica VT2100S St. Louis, MO), and processed for immunohistochemical experiments.

### 2.2. Immunohistochemistry

Immunoperoxidase staining was carried out in free-floating sections under moderate shaking conditions. After several washes in 0.1 M, pH 7.4 phosphate buffer (PB), the endogenous peroxidase activity was quenched in a solution of 1.66% hydrogen peroxide in 50% ethanol in PB for 30 min at room temperature. Thereafter, sections were preincubated for 2 h at room temperature in a stock solution containing 3% normal goat or 3% normal horse serum diluted in PB with Triton X-100 (0.3%). The sections were then incubated for 48 h at 4°C with one of the following primary antibodies: anti-SCGN (rabbit polyclonal Sigma-Aldrich Cat# Human Protein Atlas Number 006641, RRID: AB_1079874, St Louis, MO; diluted 1:1000), anti-NeuN (ABN78, rabbit polyclonal, Millipore, Billerica, MA; diluted 1:2000), anti-parvalbumin (PV 235, mouse monoclonal, Swant; diluted 1:1000) or anti-calretinin (CR6B3, mouse monoclonal, Swant, Switzerland; diluted 1:1500) in the same stock solution. After incubation with the primary antibodies, the sections were then rinsed in buffer and incubated for 2 h at room temperature with the corresponding secondary antibody: biotinylated goat anti-rabbit immunoglobulin G (BA1000, Vector laboratories, Burlingame, CA) or biotinylated horse anti-mouse immunoglobulin G (BA2000, Vector laboratories, Burlingame, CA) diluted 1:200 in PB. After several washes in buffer, the sections were incubated for 1 h at room temperature with avidin–biotin peroxidase complex (ImmunoPure ABC, Pierce, Rockford, IL; diluted 1:125). Peroxidase activity was revealed with 0.01% hydrogen peroxide, using 3,3′- diaminobenzidine (Sigma, St Louis. MO; 0.05%). Finally, the sections were mounted, dehydrated and coverslipped with DEPEX (VWR, Rannor, PA). The slides were observed with a digital microscope (Zeiss). Immunostaining was absent when the primary antibody was omitted.

For triple immunofluorescence assays, sections were first rinsed in PB and preincubated for 2 h at room temperature in a stock solution containing 3% bovine serum albumin (BSA) (A2153, Sigma-Aldrich, St Louis. MO) diluted in PB with Triton X-100 (0.3%). Thereafter, the sections were incubated for 48 h at 4°C in the same stock solution containing the following primary antibodies in combination with anti-secretagogin (rabbit polyclonal Sigma-Aldrich Cat# Human Protein Atlas Number 006641, RRID:AB_1079874, St Louis, MO; diluted 1:500): anti-parvalbumin (PV 235, mouse monoclonal, Swant; diluted 1:1000); anti-calretinin (CR6B3, mouse monoclonal, Swant, Switzerland; diluted 1:1500); anti-nNOS (ab1376, goat polyclonal, Abcam, Cambridge, United Kingdom; diluted 1:300). Furthermore, some sections were used to examine only the colocalization of PV with CR in double-immunostained sections.

The sections were then rinsed in buffer and incubated for 2 h at room temperature with biotinylated horse anti-mouse Immunoglobulin G (BA2000, Vector laboratories, Burlingame, CA; diluted 1:200). After several washes in PB, the sections were incubated for 2 h at room temperature with Streptavidin Alexa 594 (S32356, Molecular Probes, ThermoFisher Scientific, Waltham, MA; diluted 1:1000), Alexa 488 donkey anti-goat and Alexa 647 goat anti-rabbit secondary antibodies (Molecular Probes, ThermoFisher Scientific, Waltham, MA; diluted 1:1000).

For the NeuN, PV and CR triple immunofluorescence assay, free-floating sections were rinsed in PB and were blocked for 2 h at room temperature in the stock solution described above and incubated overnight at 4°C and then 1 h at room temperature in the same stock solution containing the antibody anti-calretinin (CR6B3, mouse monoclonal, Swant, Switzerland; diluted 1:1500). The sections were then rinsed in PB and incubated for 2 h at room temperature with biotinylated horse anti-mouse Immunoglobulin G (BA2000, Vector laboratories, Burlingame, CA; diluted 1:200). After several washes in PB, the sections were incubated for 2 h at room temperature with Streptavidin Alexa 594 (S32356, Molecular Probes, ThermoFisher Scientific, Waltham, MA; diluted 1:1000). Thereafter, the sections were rinsed in buffer and incubated overnight at 4°C and then 1 h at room temperature in the same stock solution containing the antibodies anti-parvalbumin (AB11427, rabbit polyclonal, Abcam, Cambridge, United Kingdom; diluted 1:1000) and anti-NeuN (ABN90, guinea pig, Millipore, Burlington, MA; diluted 1:1000). The sections were then rinsed in PB and incubated for 2 h at room temperature with Alexa 488 goat anti-rabbit and Alexa 647 goat anti-guinea pig antibodies (Molecular Probes, ThermoFisher Scientific; diluted 1:1000). In the present study, brain sections from AB7 only were employed for the PV and CR double-immunohistochemistry assay.

Finally, the slices were counterstained with DAPI (4, 6-diamidino-2-phenylindole) (Sigma, San Louis; MO; diluted 1:80). To eliminate lipofuscin autofluorescence in human samples, eliminator reagent (2,160, Millipore) was used following the manufacturer’s instructions. The sections were then mounted and coverslipped with ProLong Gold antifade reagent (Life technologies, Carlsbad, CA). To confirm the specificity for DAB and fluorescence immunostaining, negative controls were performed in parallel with the primary experiments, omitting the primary antibody under the same conditions.

### 2.3. Microscopy, quantification, and statistical analyses

Fluorescent labeling profiles in the BA10 and the BA21 were counted in stacks of fluorescence images acquired using an LSM710 confocal laser-scanning microscope (Carl Zeiss MicroImaging, Jena, Germany) and were imaged using excitation wavelengths of 488, 594, and 633 nm to visualize Alexa fluor 488, 594 and 647, respectively.

Consecutive stacks of images at low magnification (×20; zoom: 1.2; voxel size, 0.692 × 0.692 × 1 μm^3^) were acquired to capture fluorescent labeling profiles, and ImageJ software was then used to split the channels from each optical series. EspINA (Interactive Neuron Analyzer) software was employed for the colocalization studies in stacks of fluorescence images from each channel [Espina Interactive Neuron Analyzer, 2.4.1; Madrid, Spain; https://cajalbbp.es/espina/; (Morales et al., 2011)]. Firstly, fluorescent labeling profiles were individually segmented and visualized in different channels simultaneously. Secondly, we analyzed the colocalization of fluorescent labeling profiles (SCGN, PV, CR, and nNOS). The somata of labeled cells were numbered and recorded in Excel files. We counted all positive somata in columns 1,100–1,400 μm wide, throughout the whole cortical depth (from layer I to layer VI) of two 50 μm-thick sections per subject (AB1, AB2, and AB3) in BA10 and BA21. Morphological features of labeled cells were also manually measured sing EspINA software. For the statistical analysis, Fisher Exact test was performed to compare the experimental groups. The significance of the results were expressed as: *, *p*-value<0.05; **, *p*-value<0.01; ***, *p*-value<0.001; ns, no significant differences. Graphs represent mean values ± SEM.

## 3. Results

In this study, we first examined the pattern of immunoreactivity for SCGN in two areas of the neocortex: Brodmann’s area 10 (BA10; also known as the frontal pole or rostral/anterior prefrontal cortex) and Brodmann’s area 21 (BA21; also known as the middle temporal cortex; see Zilles and Amunts, 2010). The cytoarchitectonic characteristics of the cortical regions examined were based on Nissl and DAPI staining, with NeuN-immunostained sections also used. In both BA10 and BA21 areas, six major cortical layers can be readily identified. Layers I and II have similar widths in both areas. The widest layer is III, and we have subdivided this layer into two sublayers: IIIa and IIIb. Layer IV has clear borders with the layers above (III) and below (V), and it is relatively thin. Layer V is wider than layer IV, whereas layer VI is wider than layer V. Layers V and VI were not further subdivided.

The second principal goal was to investigate the degree of immunocytochemical colocalization of SCGN with the calcium-binding proteins parvalbumin (PV) and calretinin (CR) as well as with neuronal nitric oxide synthase (nNOS), the enzyme responsible for the synthesis of nitric oxide (NO) by neurons.

### 3.1. Pattern of SCGN immunostaining in the human frontal (BA10) and temporal (BA21) cortex

**Figure 1:**
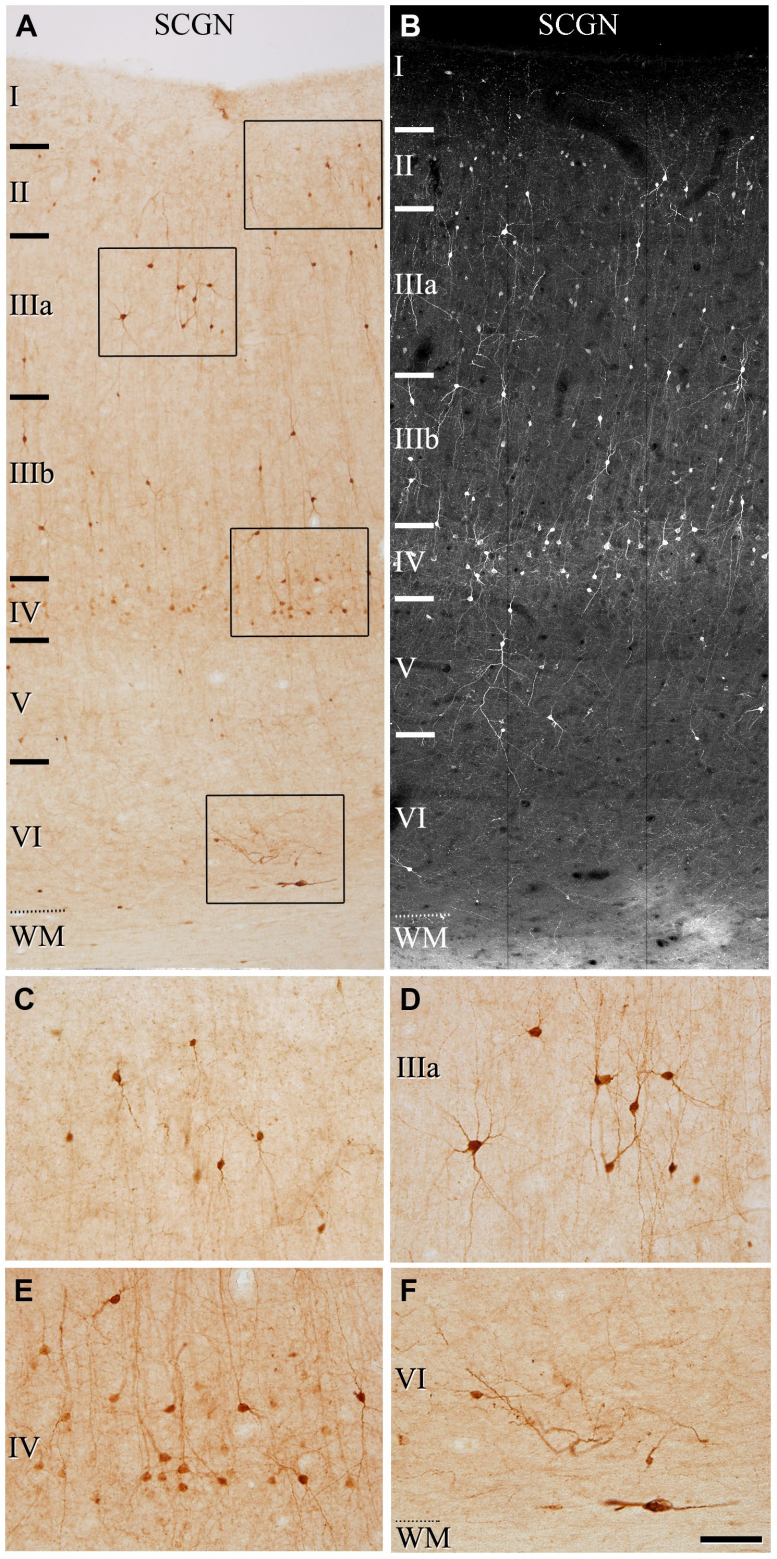
Photomicrographs from a representative section illustrating the pattern of SCGN staining in the frontal cortex (BA10) in a 50 μm-thick DAB-immunostained section **(A)** and in a fluorescence immunostained section (confocal projection image from a z-stack of images corresponding to a z-thickness of 33 μm) **(B)**. Rectangles in **(A)** indicate regions shown at a higher magnification in **(C–F)**. In **(A,B)**, note the non-homogeneous distribution of the immunoreactivity pattern of SCGN+, with the highest density of cells in layer IV. Scale bar shown in **(F)** indicates 150 μm in **(A,B)**, and 70 μm in **(C–F)**. Cortical layers, I–VI; WM, white matter. Adobe Photoshop CS4 (Adobe Inc., 2019) software was used to compose figures.

**Figure 2:**
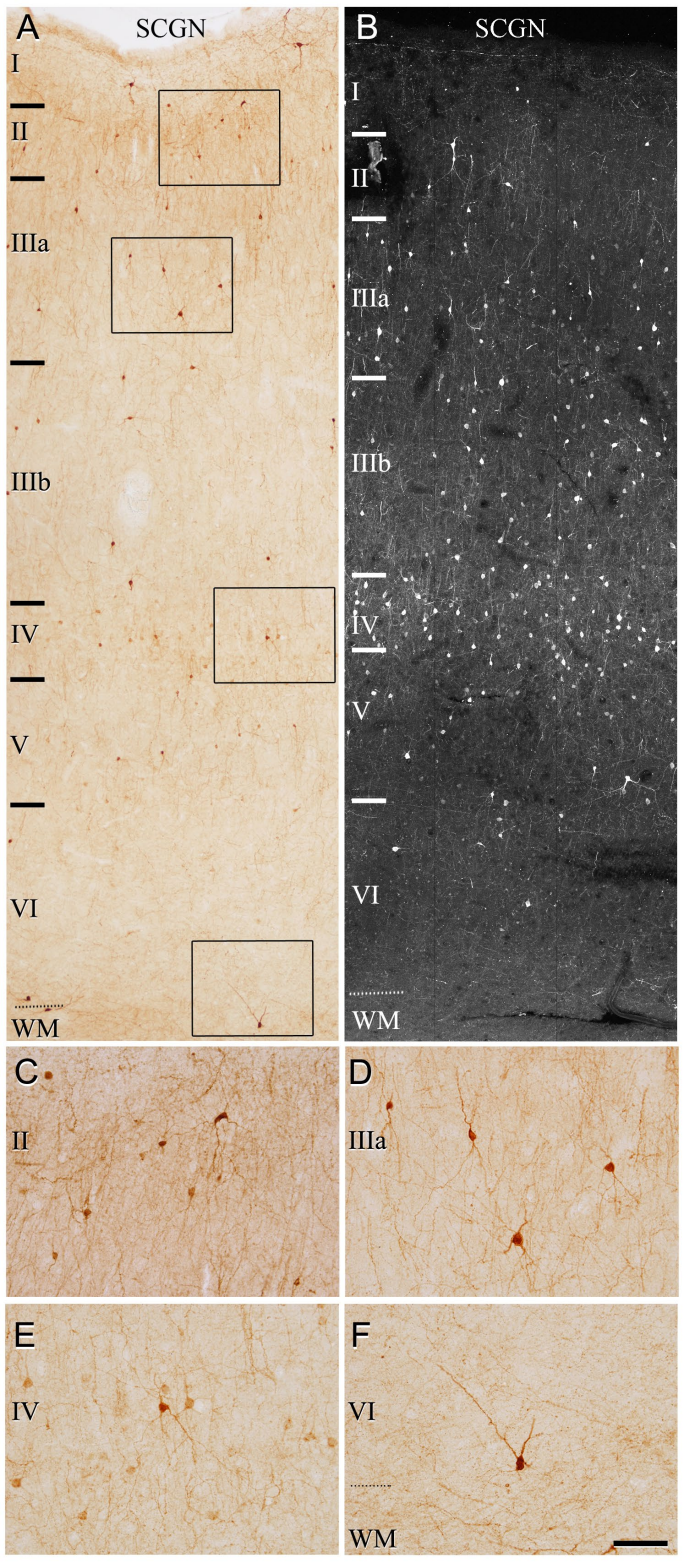
Photomicrographs from a representative section illustrating the pattern of SCGN staining in the temporal cortex (BA21) in a 50 μm-thick DAB-immunostained section **(A)** and in a fluorescence immunostained section (confocal projection image from a z-stack of images corresponding to a z-thickness of 33 μm) **(B)**. Rectangles in **(A)** indicate regions shown at a higher magnification in **(C–F)**. Note the non-homogeneous distribution of the immunoreactivity pattern of SCGN+ cells, with the highest density of cells in layer IV. Scale bar shown in **(F)** indicates 150 μm in **(A,B)** and 70 μm in **(C–F)**. Cortical layers, I–VI; WM, white matter. Adobe Photoshop CS4 (Adobe Inc., 2019) software was used to compose figures.

We first assessed the distribution pattern of SCGN-immunoreactive neurons (SCGN+) through all the cortical layers (layers I, II, IIIA, IIIB, IV, V, and VI). SCGN+ cells were present throughout all layers in both BA10 (Figure 1) and BA21 (Figure 2), but they were not homogeneously distributed; they were more frequently observed in layers II, III and IV, whereas in layer I they were found only occasionally. In general, the labeled neuropil showed a light diffuse staining in which scattered stained neuronal processes are distinguished. As previously described in our study of the hippocampal formation and adjacent cortex (Tapia-Gonzalez et al., 2020), we observed two main types of immunostained cells based on the intensity of labeling (Figures 1C–F, 2C–F): (1) SCGN-type I cells, characterized by a dark brown (Golgi-like) staining in DAB-immunostained sections or strong labeling in fluorescence-immunostained sections and (2) SCGN-type II cells, characterized by a light brownish-gray staining in DAB-immunostained sections or light labeling in fluorescence-immunostained sections. All the labeled neurons exhibited a non-pyramidal morphology (interneurons).

The most frequent morphological types of SCGN+ cells were bipolar, bitufted and multipolar cells. Bipolar cells had round or fusiform somata which gives rise to two vertically oriented primary dendrites, whereas bitufted and multipolar cells had large fusiform or polygonal somata, respectively, which give rise to several dendrites (Figures 1C–F, 2C–F). The diameters of the soma of these cells ranged from 7 to 17 μm (the total number of cells examined including all layers was 280 cells in BA10 and 210 in BA21).

We also observed neurons in the white matter that were immunoreactive for SCGN, below the boundary with layer VI (“true white matter” see Garcia-Marin et al., 2010).

### 3.2. Pattern of PV and CR immunostaining in the human frontal (BA10) and temporal (BA21) cortex

**Figure 3:**
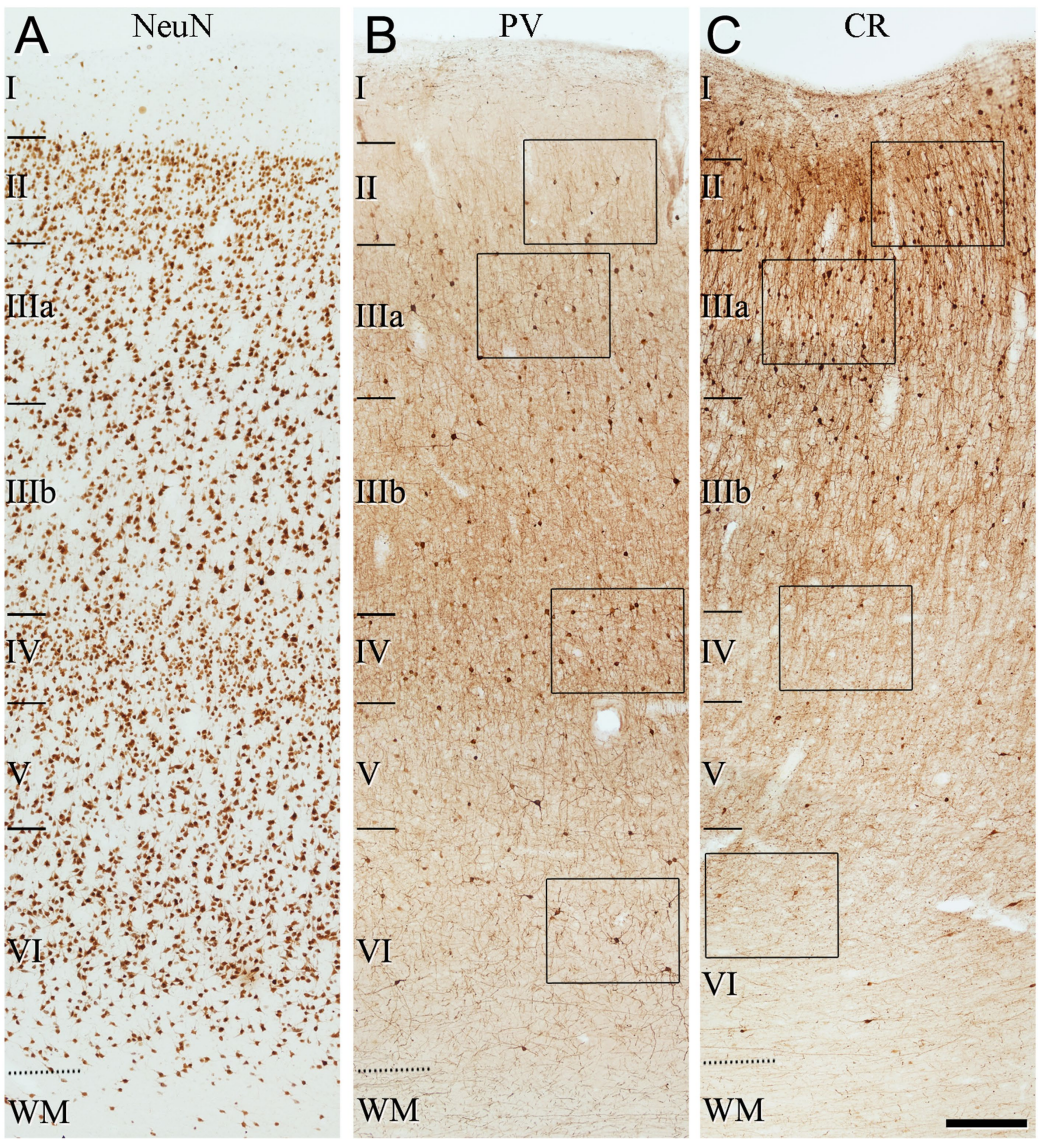
Low-magnification photomicrographs illustrating the pattern of immunostaining for NeuN **(A)**, PV **(B)** and CR **(C)**, throughout all layers of the frontal cortex (BA10). The areas indicated by rectangles in **(B,C)** are shown at a higher magnification in Figure 4. Note in **(B)** that PV+ cells are mainly observed in layer II (close to the boundary with layer IIIa), and layers IIIa, IIIb and IV. By contrast, CR+ cells are found mainly in superficial layers I–IIIb. Scale bar shown in **(C)** indicates 240 μm in all panels. Cortical layers, I–VI; WM, white matter. Adobe Photoshop CS4 (Adobe Inc., 2019) software was used to compose figures.

**Figure 4:**
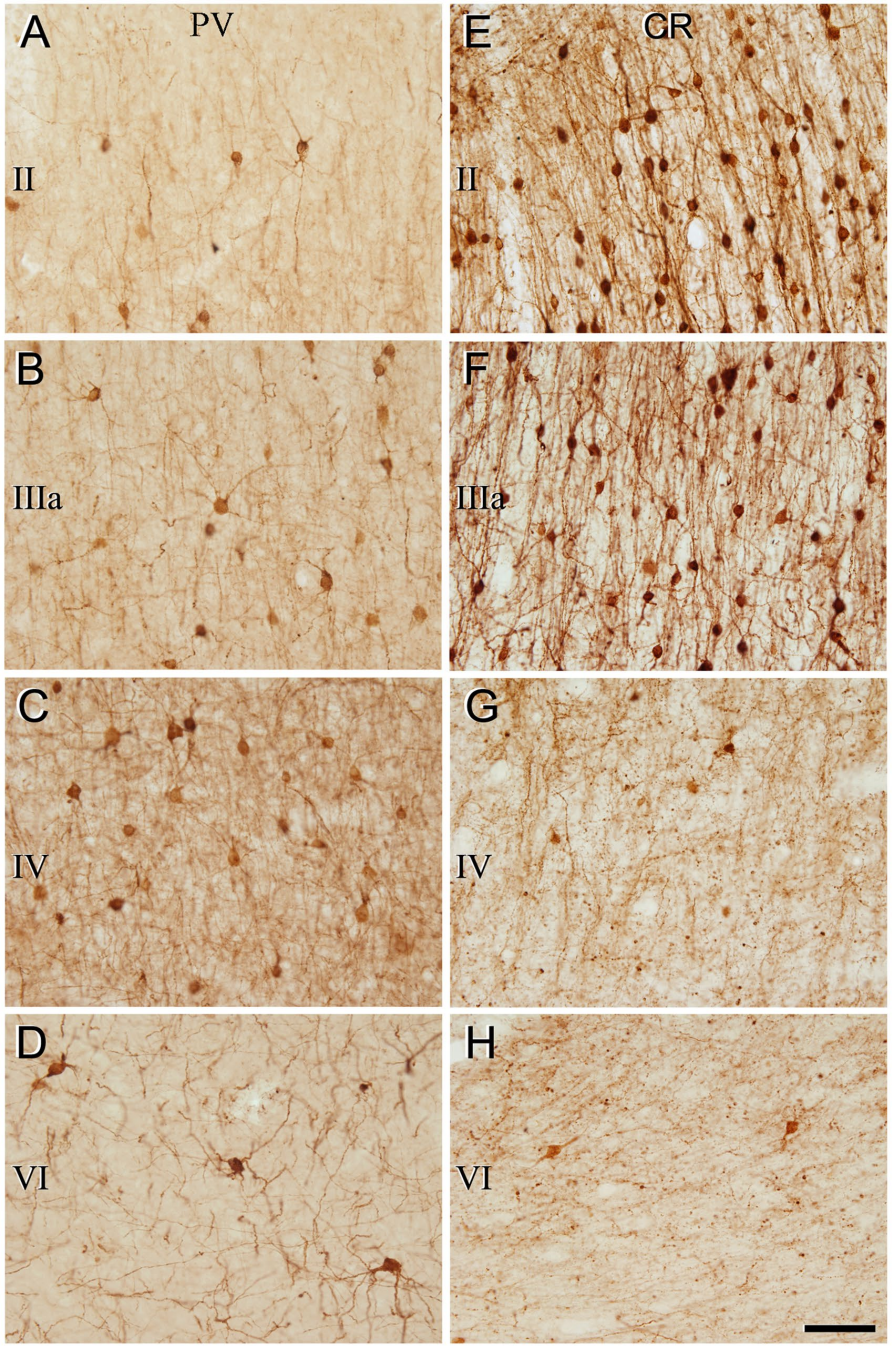
Higher magnification of the areas indicated by rectangles in Figure 3 (frontal cortex, BA10). **(A–H)** Neurons labeled in layers II, IIIa, IV, and VI for PV **(A–D)** and for CR **(E–H)**. Note the differences in the density of immunostained neurons for PV and CR in the different cortical layers. Scale bar shown in **(H)** indicates 70 μm in all panels. Cortical layers, I–VI. Adobe Photoshop CS4 (Adobe Inc., 2019) software was used to compose figures.

**Figure 5:**
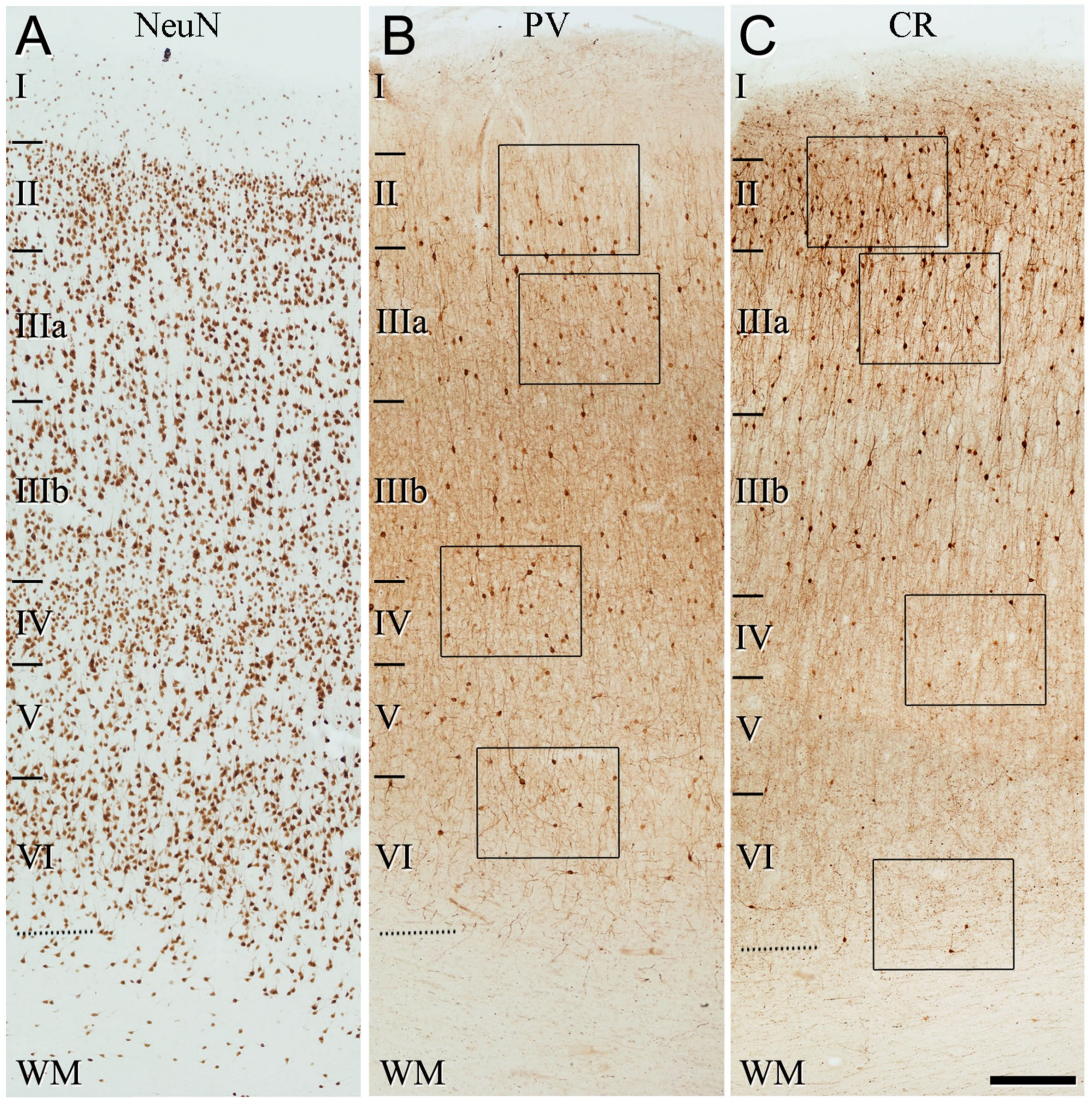
Low-magnification photomicrographs illustrating the pattern of immunostaining for NeuN **(A)**, PV **(B)**, and CR **(C)**, throughout all layers of the temporal cortex (BA21). The areas indicated by rectangles in **(B,C)** are displayed at a higher magnification in Figure 6. Note that the pattern of immunostaining is similar to that found in BA10 (Figure 3). That is, PV+ cells are mainly present in layer II (close to the boundary with layer IIIa), and in layers IIIa, IIIb, and IV, whereas CR+ cells are observed mainly in superficial layers I–IIIb. Scale bar shown in **(C)** indicates 240 μm in all panels. Cortical layers, I–VI; WM, white matter. Adobe Photoshop CS4 (Adobe Inc., 2019) software was used to compose figures.

**Figure 6:**
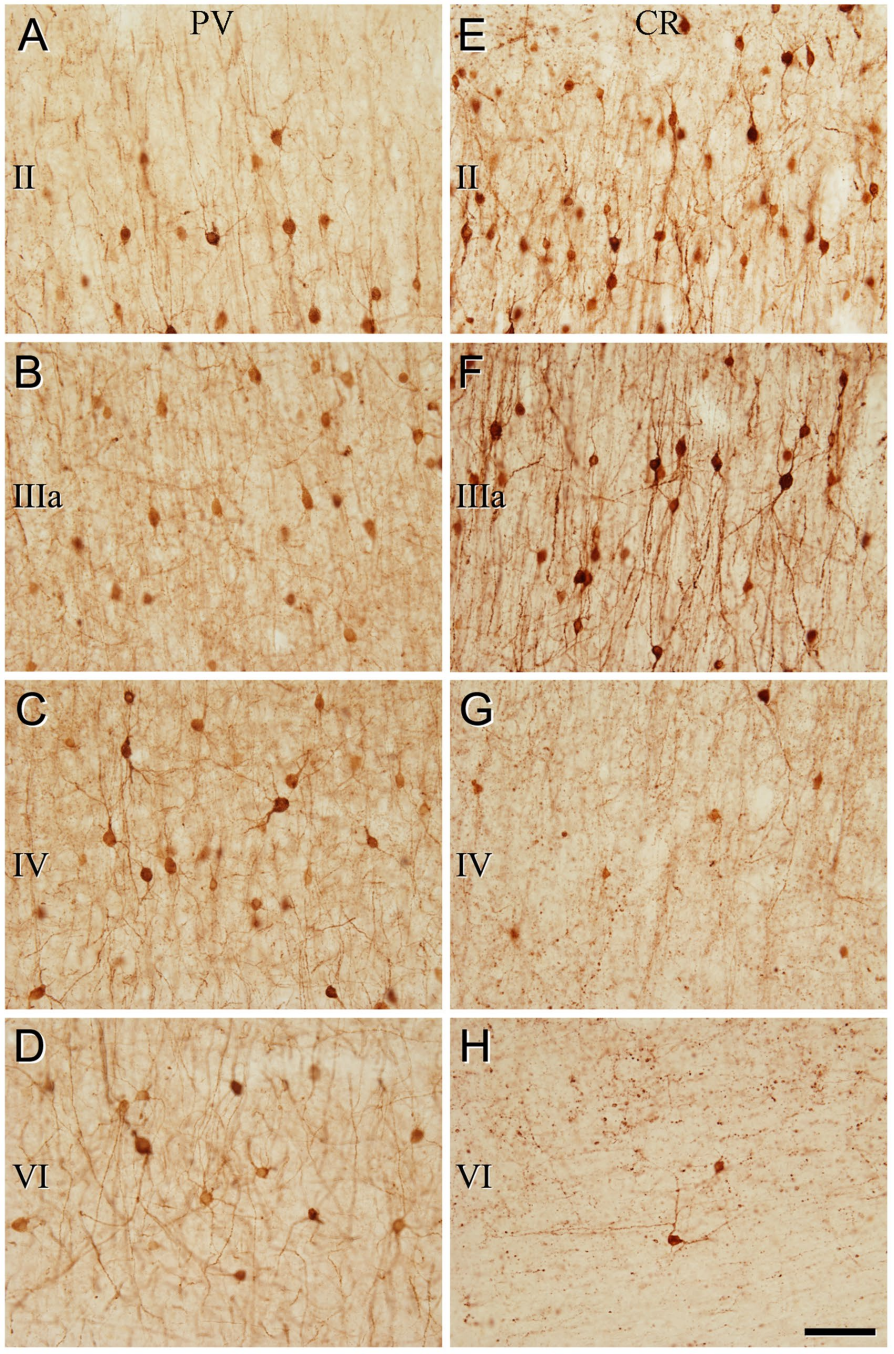
Higher magnification of the areas indicated by rectangles in Figure 5 (temporal cortex, BA21). **(A–H)** Note the differences in the density of immunostained neurons for PV and CR in the different cortical layers. Scale bar shown in **(H)** indicates 70 μm in all panels. Adobe Photoshop CS4 (Adobe Inc., 2019) software was used to compose figures.

The general pattern of immunostaining for PV and CR in the BA10 and BA21 has been described in previous studies (Conde et al., 1994; del Rio and DeFelipe, 1997; Gonzalez-Albo et al., 2001). However, to facilitate the comparison of SCGN immunostaining with PV and CR in the same brain tissue, what follows is a brief description of the pattern of immunostaining for PV and CR throughout BA10 and BA21 in adjacent sections to those used for SCGN immunostaining. In both cortical areas, PV+ neurons were mostly distributed through layers II-VI, although they were more prevalent in layer II, particularly close to the boundary with layer IIIa, and especially in layers IIIa, IIIb, and IV (Figures 3–6). By contrast, CR+ neurons were observed throughout all layers of the BA10 and the BA21, mainly in layers II, IIIa, and IIIb (Figures 3–6).

### 3.3. Colocalization of SCGN+ with PV+, CR+, and nNOS+ neurons in BA10 and BA21: morphological aspects

**Figure 7:**
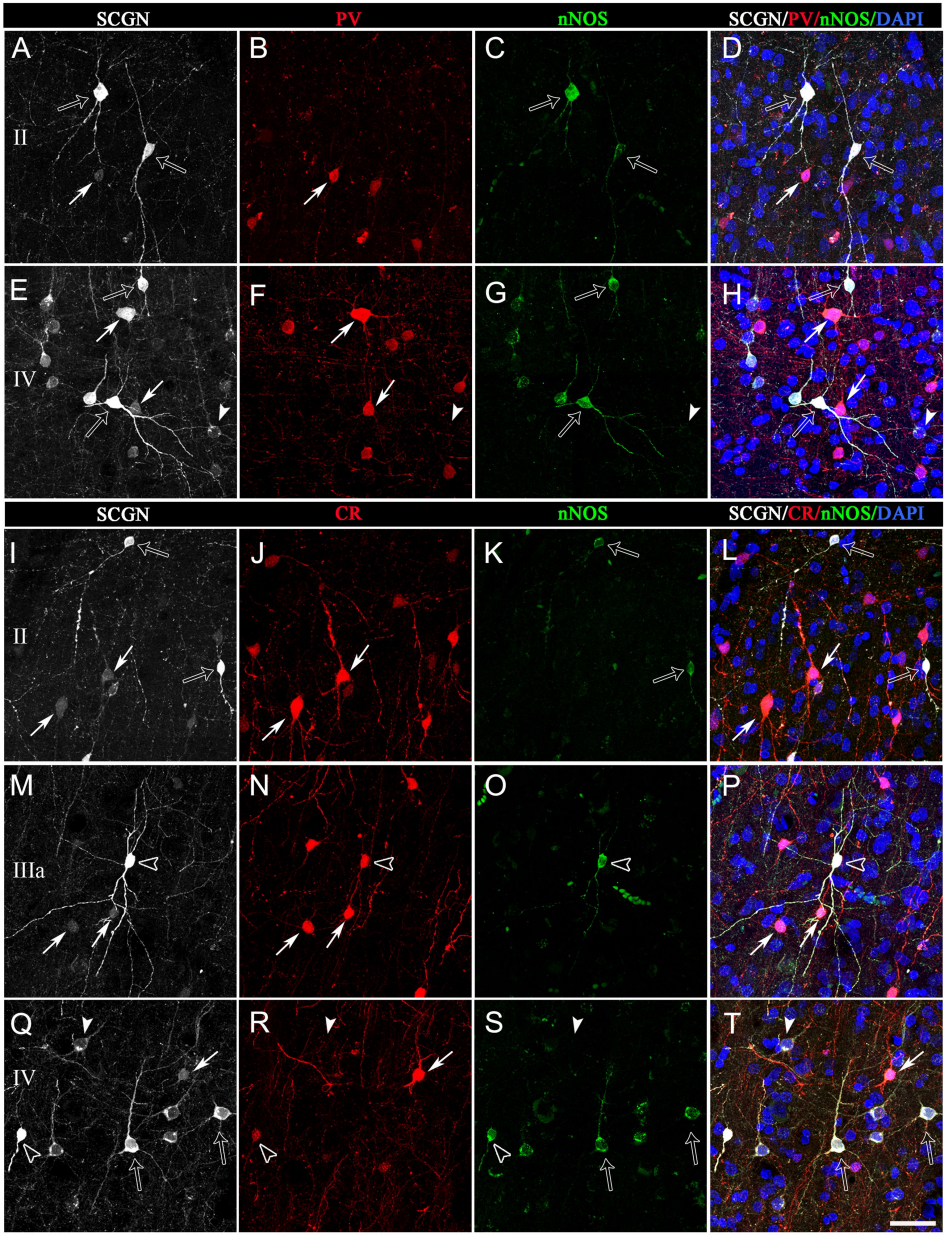
Trios of confocal projection images (z-stack of images, 12 μm) from the frontal cortex (BA10) taken from triple-immunostained sections for SCGN (white), PV (red) and nNOS (green) **(A−H)** and for SCGN (white), CR (red) and nNOS (green) **(I–P)**. Right column images **(D,H,I,P,T)** were obtained after combining images of the left panels and counterstaining with DAPI. In **(A−H)**, solid arrows indicate SCGN+/PV+ but not nNOS+ cells; open arrows, SCGN+/nNOS+ cells but not PV+; and solid arrowheads, SCGN+ not PV+ or nNOS+. In **(I–T)**, solid arrows indicate SCGN+/CR+ but not nNOS+ cells; open arrows, SCGN+/nNOS+ cells but not CR+; solid arrowheads, SCGN+ but not CR+ or nNOS+; and open arrowheads, SCGN+/CR+/nNOS+. Note the variety of morphologies of SCGN-ir cells. Scale bar shown in **(T)** indicates 40 μm in all panels. Adobe Photoshop CS4 (Adobe Inc., 2019) software was used to compose figures.

**Figure 8:**
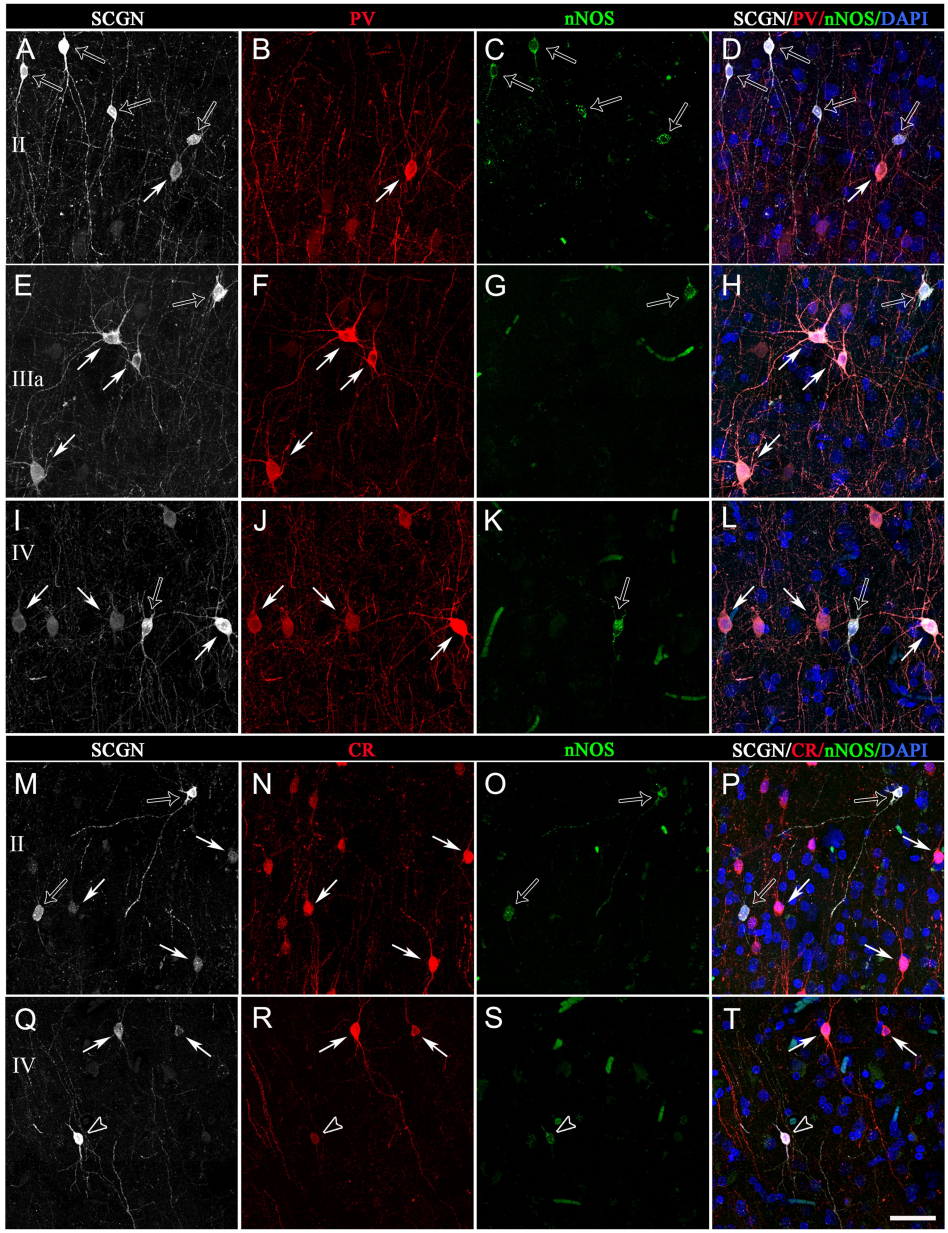
Trios of confocal projection images (z-stack of images, 12 μm) from the temporal cortex (BA21) taken from triple-immunostained sections for SCGN (white), PV (red) and nNOS (green) **(A−L)** and for SCGN (white), CR (red) and nNOS (green) **(I–P)**. The images in the right column **(D,H,I,P,T)** were obtained after combining images of the left panels and counterstaining with DAPI. In **(A–L)**, solid arrows indicate SCGN+/PV+ but not nNOS+ cells; open arrows, SCGN+/nNOS+ cells but not PV+. In **(M–T)**, solid arrows indicate SCGN+/CR+ but not nNOS+ cells; open arrows, SCGN+/nNOS+ cells but not CR+; and open arrowheads, SCGN+/CR+/nNOS+. Note the variety of morphologies of SCGN-ir cells. Scale bar shown in **(T)** indicates 40 μm in all panels. Adobe Photoshop CS4 (Adobe Inc., 2019) software was used to compose figures.

SCGN+/PV+ cells: The morphology of these double-labeled neurons was mostly multipolar with round or polygonal somata (Figures 7, 8). Bitufted cells were also found mostly in layers V-VI. The diameters of SCGN+/PV+ somata ranged from 7 to 17 μm (*n* = 70).

SCGN+/CR+ cells: The morphology of neurons was varied – from multipolar with a round soma to bipolar and bitufted morphologies with round or fusiform somata (Figures 7, 8). The diameters of the somata of SCGN+/CR+ cells ranged from 7 to 12 μm, although the vast majority of somata were around 9 to 10 μm (*n* = 70).

SCGN+/nNOS+ cells: The morphology of these neurons was also heterogeneous, with bipolar or bitufted dendritic morphologies with round or fusiform somata, or multipolar morphology with round, fusiform or polygonal somata (Figures 7, 8). The diameter of somata of SCGN+/nNOS+ cells ranged from 7 to 13 μm, although the most frequent size was 7 to 8 μm (*n* = 70). Furthermore, as previously described in the human cerebral cortex (e.g., Benavides-Piccione and DeFelipe, 2003), we observed two main types of immunolabeling for nNOS: nNOS-type I cells which displayed an intense immunolabeling, and nNOS-type II cells which were weakly stained. Both types exhibited a variety of morphological shapes (Figures 7, 8).

Finally, when considering the triple-labeled cells, the morphologies of these cells were in general less heterogeneous:

SCGN+/CR+/nNOS+: These triple-labeled cells were observed mainly in layers II–V of the BA10, and in layers II–IV of the BA21 (Figures 7, 8). These cells frequently had bitufted or multipolar shapes with round somata. The diameter of the somata of SCGN+/CR+/nNOS+ cells were from 7 to 13 μm, although the most frequent size was 7 to 10 μm (*n* = 70). As we will see below, no SCGN+/PV+/nNOS+ cells were found either in BA10 or in BA21.

### 3.4. Colocalization of SCGN+ with PV+, CR+, and nNOS+ neurons in BA10 and BA21: percentage of colocalization

The percentage of colocalization was estimated by counting the number of all labeled cells through columns (1,100–1,400 μm wide) across the entire thickness of the cortex (from the pial surface to the white matter) in 50 μm-thick sections from both BA10 and BA21. Several hundred labeled cells from layers I to VI for each antigen were analyzed to estimate the percentage of colocalization between different antigens in individual neurons (Tables 1, 2). As shown in Supplementary Figures S1, S2, there was relatively little variability between the data obtained in the three cases studied. Therefore, the colocalization results collectively obtained in the three human cases are described here. All possible combinations of SCGN+ cells expressing PV and/or nNOS, as well as SCGN+ cells expressing CR and/or nNOS, were observed in different proportions in all layers of both cortical areas, except for the subpopulation SCGN+/PV+/nNOS+ because no SCGN+ neurons were observed that expressed both PV and nNOS.

**Table 1:** Number and percentage (mean ± SEM) of SCGN+ neurons immunostained or not for PV and/or nNOS in all layers of BA10 and BA21.

Subpopulation SCGN^+^ cells	*N*° cells	% cells	% cells/layer					
			LI	LII	LIII	LIV	LV	LVI
**BA10**								
**SCGN+/PV+/**nNOS−	1,043	63.14 ± 5.88	0.00 ± 0.00	46.68 ± 2.56	66.09 ± 1.49	63.57 ± 7.30	78.12 ± 3.47	44.02 ± 9.48
**SCGN+/**PV−**/**nNOS−	400	23.78 ± 1.64	26.67 ± 4.37	19.99 ± 6.69	20.32 ± 1.47	28.68 ± 4.04	16.06 ± 1.67	38.78 ± 6.79
**SCGN+/nNOS+/**PV−	206	13.07 ± 4.26	73.33 ± 4.37	33.34 ± 6.20	13.58 ± 2.80	7.75 ± 3.56	5.81 ± 3.17	17.21 ± 2.96
**BA21**								
**SCGN+/PV+/**nNOS−	1,021	70.59 ± 5.30	0.00 ± 0.00	51.92 ± 3.68	72.35 ± 3.42	81.67 ± 4.21	75.93 ± 1.91	78.04 ± 1.04
**SCGN+/**PV−**/**nNOS−	292	24.62 ± 4.63	95.83 ± 3.40	35.88 ± 3.98	24.85 ± 3.75	14.73 ± 4.22	17.28 ± 3.02	13.48 ± 1.82
**SCGN+/nNOS+/**PV−	60	4.79 ± 0.99	4.17 ± 3.40	12.20 ± 2.17	2.80 ± 0.34	3.61 ± 1.80	6.79 ± 1.14	8.48 ± 1.76

**Table 2:** Number and percentage (mean ± SEM) of SCGN+ neurons immunostained or not for CR and/or nNOS in all layers of BA10 and BA21.

Subpopulation SCGN+ cells	*N*° cells	% cells	% cells/layer					
			LI	LII	LIII	LIV	LV	LVI
**BA10**								
**SCGN+/CR+/**nNOS−	325	40.05 ± 2.19	66.95 ± 4.98	55.46 ± 5.15	56.01 ± 5.56	11.97 ± 3.44	9.08 ± 3.71	40.63 ± 4.16
**SCGN+/**CR−**/**nNOS−	324	41.48 ± 4.37	10.83 ± 4.76	26.94 ± 7.16	31.64 ± 6.53	51.45 ± 2.98	66.64 ± 3.39	44.67 ± 4.96
**SCGN+/nNOS+/**CR−	132	13.01 ± 2.38	22.22 ± 4.09	8.91 ± 0.74	7.40 ± 0.70	29.51 ± 2.16	19.91 ± 4.05	14.70 ± 1.71
**SCGN+/CR+/nNOS+**	44	5.46 ± 0.21	0.00 ± 0.00	8.69 ± 2.25	4.95 ± 0.68	7.07 ± 1.42	4.38 ± 2.41	0.00 ± 0.00
**BA21**								
**SCGN+/CR+/**nNOS−	584	59.41 ± 5.27	40.84 ± 6.97	71.51 ± 5.48	70.45 ± 3.93	25.72 ± 7.38	11.11 ± 4.54	36.67 ± 10.89
**SCGN+/**CR−**/**nNOS−	228	26.14 ± 3.82	42.92 ± 3.64	16.60 ± 4.96	15.41 ± 1.48	59.34 ± 4.36	55.30 ± 2.17	49.44 ± 7.47
**SCGN+/nNOS+/**CR−	83	10.53 ± 1.38	16.24 ± 4.32	8.68 ± 0.71	8.87 ± 2.02	8.43 ± 3.56	33.59 ± 3.75	13.89 ± 6.00
**SCGN+/CR+/nNOS+**	25	3.92 ± 0.12	0.00 ± 0.00	3.21 ± 0.92	5.27 ± 2.10	6.52 ± 0.51	0.00 ± 0.00	0.00 ± 0.00

In Table 1, the percentage of SCGN+ neurons immunostained or not for PV and/or nNOS is presented. As shown in this table, in BA10, the largest subpopulation of SCGN+ cells expressed PV but not nNOS (SCGN+/PV+/nNOS−) (approximately 61%), followed by the subpopulation of SCGN+ cells that were not labeled for PV or nNOS (SCGN+/PV−/nNOS−) (25%), and SCGN+ cells that were also immunostained for nNOS but not for PV (SCGN+/PV−/nNOS+) (14%). In BA21, the percentages of colocalization were similar except for the expression of nNOS, which was lower than in BA10. These percentages, from highest to lowest, were as follows: SCGN+/PV+/nNOS− (72%), SCGN+/PV−/nNOS− (23%) and (SCGN+/PV−/nNOS+) (5%).

**Figure 9:**
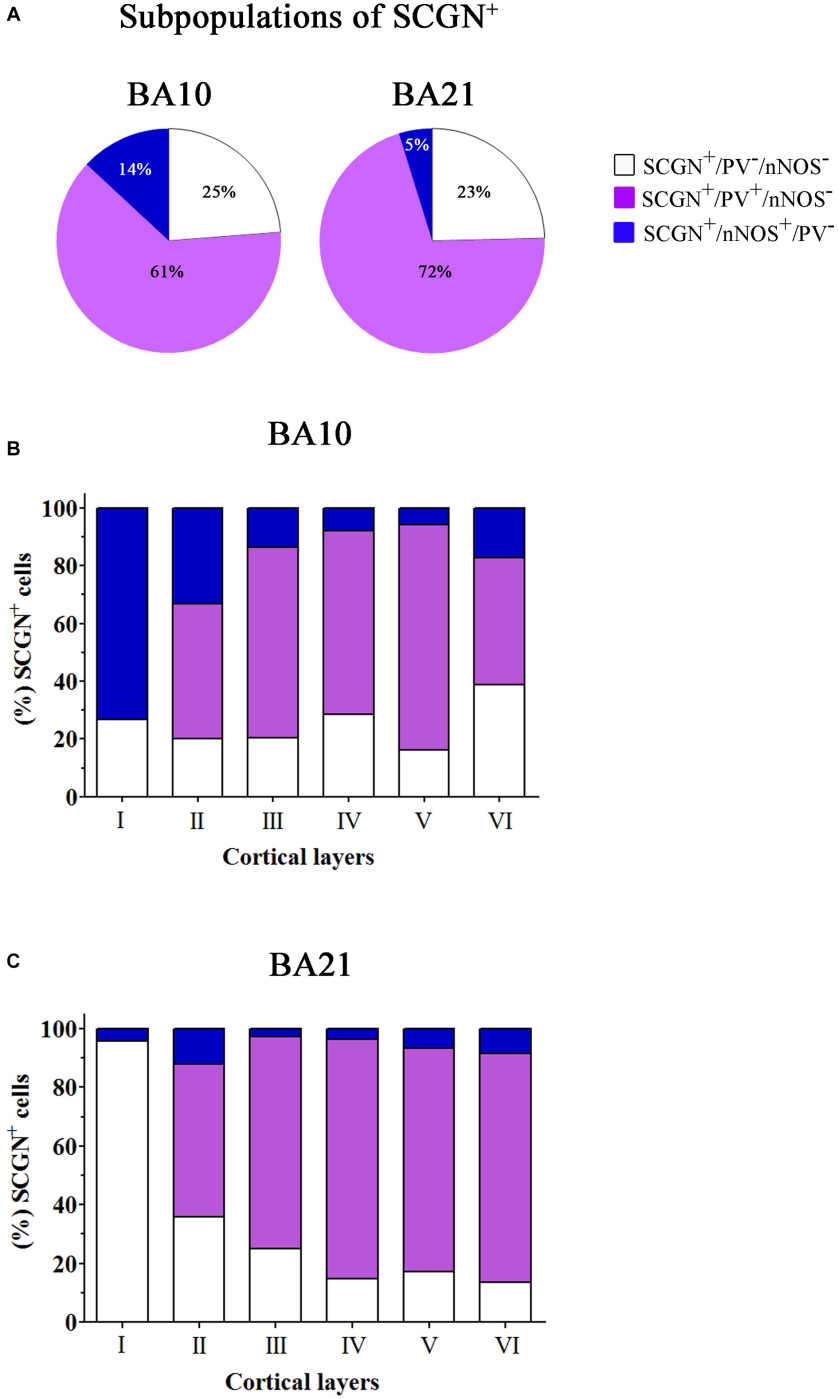
Percentage of colocalization of SCGN+ cell also immunostained for PV or nNOS through columns (1100–1,400 μm wide) across the entire thickness of the cortex (from the pial surface to the white matter) in 50 μm-thick sections from both BA10 and BA21. **(A)** Pie charts showing the percentage of SCGN+ cells that do not express PV or nNOS (white) and the percentage of colocalization of SCGN+ neurons with PV (violet) or nNOS (blue). **(B,C)** Graphs showing the distribution of different SCGN+ subpopulations across the cortical layers in BA10 **(B)** and BA21 **(C)**. Note that the majority of SCGN+ cells colocalized with PV and that the smallest subpopulation of SCGN+ cells are those also labeled for nNOS, particularly in BA10. Also note the differences per cortical layer in the colocalization of SCGN+ cells with PV and nNOS. Measurements are reported as mean ± SEM (see Table 1). The statistical significance of these differences is shown in Supplementary Tables S1, S3. There were significant differences in the percentage of colocalization of SCGN+ cells with PV and nNOS between certain cortical layers.

**Figure 10:**
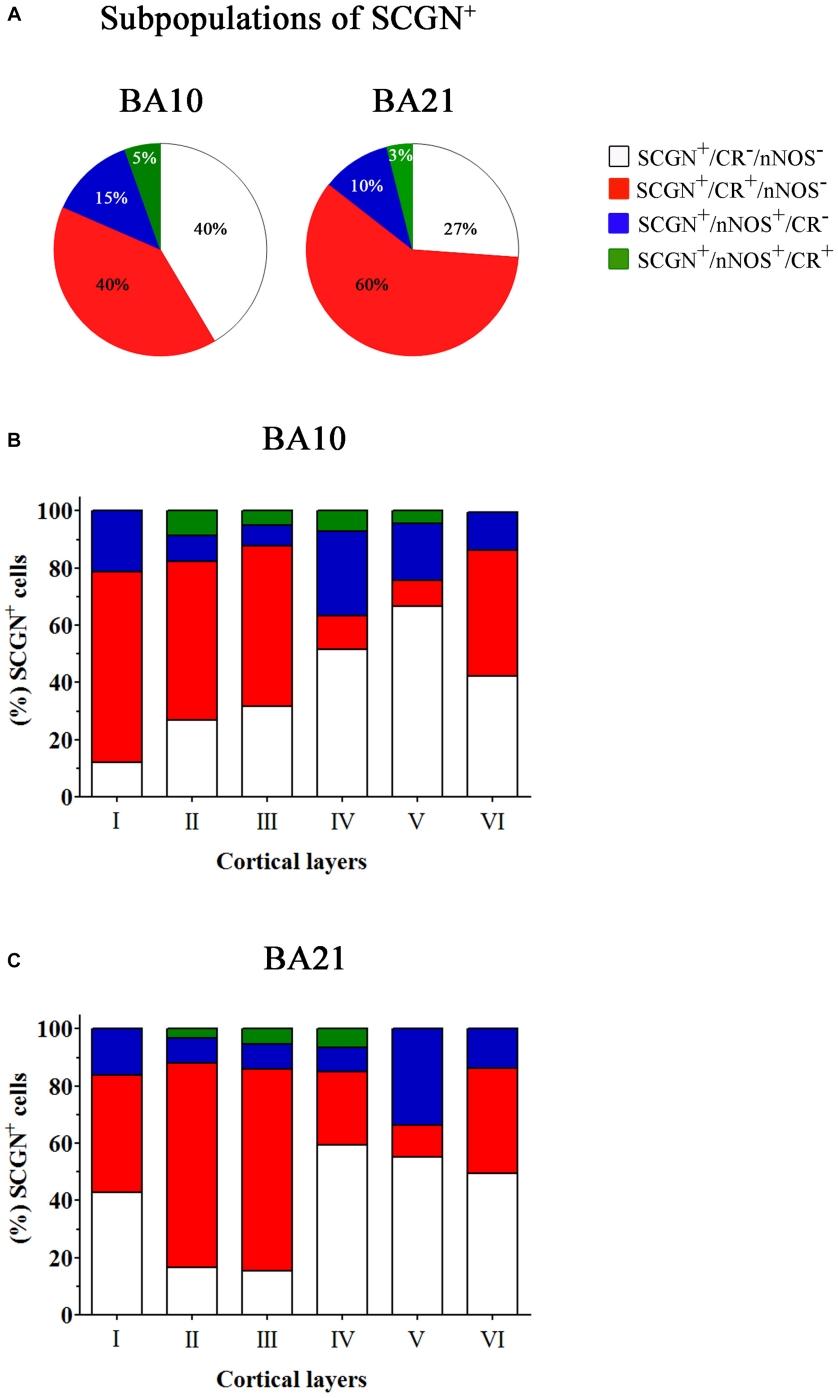
Percentage of SCGN+ cells also immunostained for CR or nNOS – or for both CR and nNOS – through columns (1100–1,400 μm wide) across the entire thickness of the cortex (from the pial surface to the white matter) in 50 μm-thick sections from both BA10 and BA21. **(A)** Pie charts showing the percentage of SCGN+ cells that do not express CR or nNOS (white), the percentage of colocalization of SCGN+ neurons with CR (red) or nNOS (blue), and with both CR and nNOS (green). **(B,C)** Graphs showing the distribution of different SCGN+ subpopulations across the cortical layers in BA10 **(B)** and BA21 **(C)**. Note the differences per cortical layer in the colocalization of SCGN+ cells with CR or nNOS and with both CR and nNOS. Measurements are reported as mean ± SEM (see Table 2). The statistical significance of the differences is shown in Supplementary Tables S2, S4. There were significant differences in the percentage of colocalization of SCGN+ cells with CR and between certain cortical layers.

In Table 2, the percentage of SCGN+ neurons immunostained or not for CR and/or nNOS is presented. As shown in this table, in BA10 we observed two large subpopulations of SCGN+ neurons: SCGN+ cells that did not express CR or nNOS (approximately 40%) (SCGN+/CR−/nNOS−) and SCGN+ cells that also expressed CR but not nNOS (40%) (SCGN+/CR+/nNOS−). The percentage of other subpopulations was relatively low: 15% of SCGN+ cells were also labeled for nNOS but not for CR (SCGN+/nNOS+/CR−), and 5% of SCGN+ cells were also immunostained for CR and nNOS (SCGN+/CR+/nNOS+). In BA21, the percentages of colocalization were similar except for the expression of CR+, which was higher than in BA10. These percentages, from highest to lowest, were SCGN+/CR+/nNOS− (60%), SCGN+/CR−/nNOS− (27%), SCGN+/nNOS+/CR− (10%) and SCGN+/CR+/nNOS+ (3%) (Figures 9, 10).

Statistical comparisons of mean percentages of different subpopulations of SCGN+ cells throughout layers I–VI in BA10 and BA21 are shown in Supplementary Tables S1–S4. As shown in these Supplementary Tables, significant differences in the percentage of colocalization of SCGN+ cells with PV, CR and nNOS were observed between certain cortical layers.

### 3.5. Other colocalization studies

**Figure 11:**
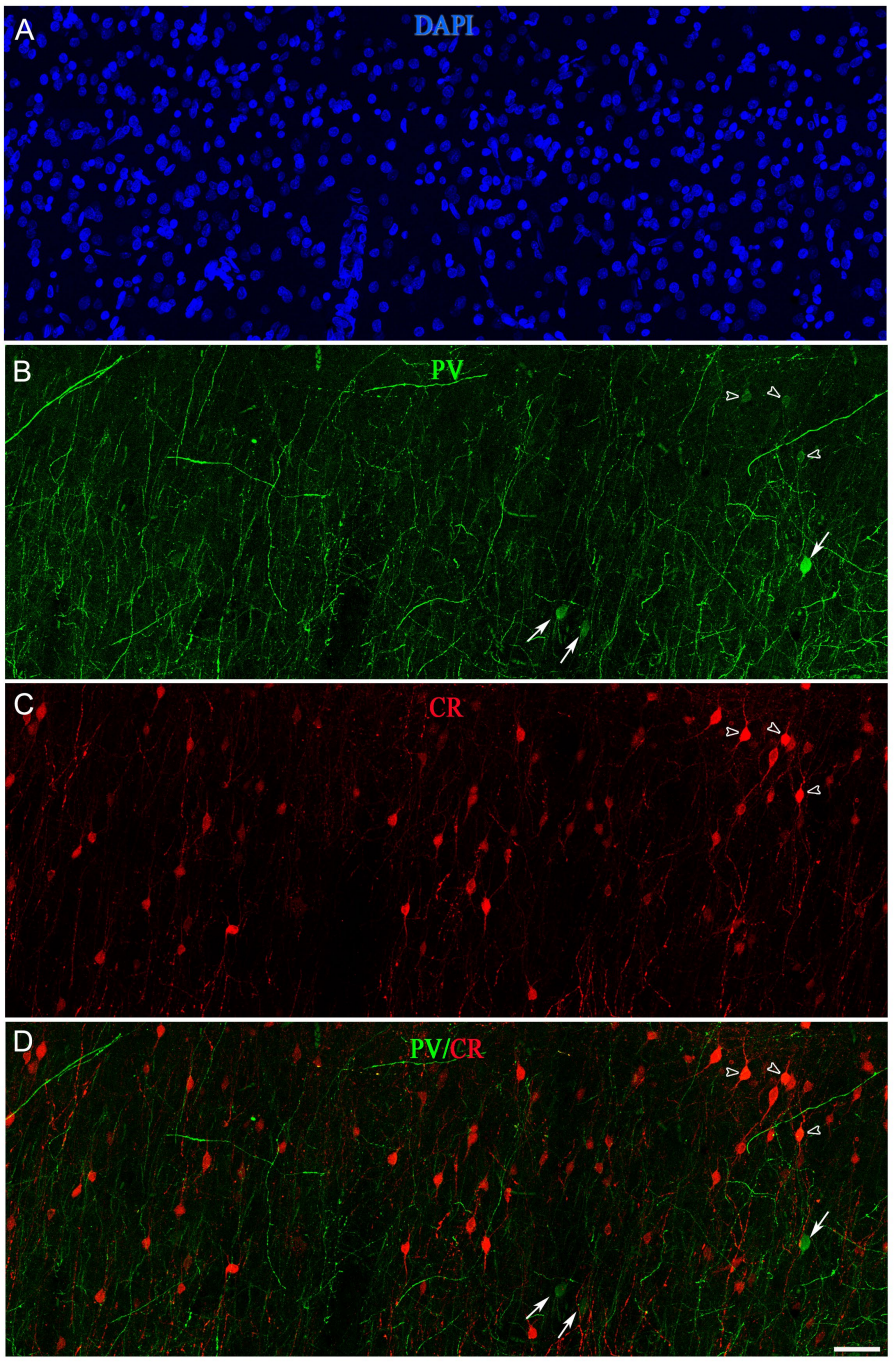
Confocal projection images (z-stack of images, 17 μm) from layer II of the frontal cortex (BA10) taken from a double-immunostained section for PV (green) **(B)** and CR (red) **(C)**, and counterstained with DAPI (blue) **(A)**. **(D)** Image obtained after combining **(B,C)**. Open arrowheads indicate PV+/CR+ cells; solid arrows show PV+ but not CR+. Scale bar shown in **(D)** indicates 45 μm in all panels. Adobe Photoshop CS4 (Adobe Inc., 2019) software was used to compose figures.

**Figure 12:**
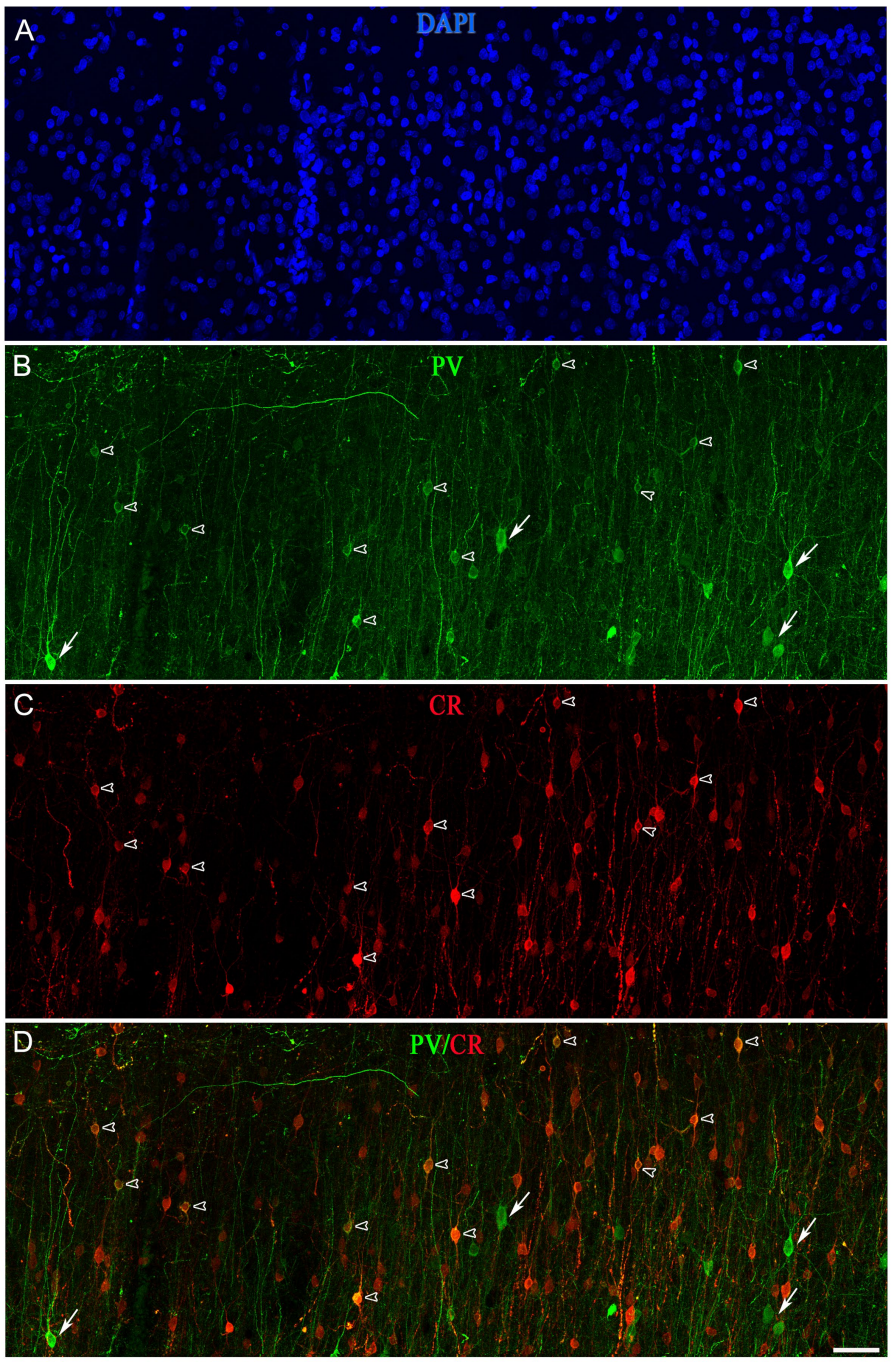
Projection confocal images (z-stack of images, 17 μm) from layer II of the temporal cortex (BA21) taken from a double-immunostained sections PV (green) **(B)** and CR (red) **(C)**, and counterstained with DAPI (blue) **(A)**. **(D)** Image obtained after combining **(B,C)**. Open arrowheads indicate PV+/CR+ cells; solid arrows show PV+ but not CR+. Scale bar shown in **(D)** indicates 45 μm in all panels. Adobe Photoshop CS4 (Adobe Inc., 2019) software was used to compose figures. Adobe Photoshop CS4 (Adobe Inc., 2019) software was used to compose figures.

A subpopulation of SCGN+ cells did not display colocalization with PV, CR or nNOS, whereas a high percentage of SCGN+/PV+/nNOS− and SCGN+/CR+/nNOS− cells were found in both BA10 and BA21 (Tables 1, 2). To examine whether or not the subpopulation of SCGN+/PV+/nNOS− and SCGN+/CR+/nNOS− represent overlapping subpopulations, we examined brain sections that were processed for triple immunocytochemical staining for SCGN, PV, and CR. Unfortunately, this immunocytochemical combination did not work for us. Thus, we examined this issue indirectly by performing colocalization experiments of PV with CR in both BA10 and BA21. Since the majority of CR+ neurons are found in layers II and IIIa–IIIb (see above), we focused on these layers. A total of 411 PV+ cells in BA10 and 601 PV+ cells in BA21 were examined in these experiments. We found that PV+ neurons that were also CR+ represented approximately 8% of the PV+ neurons in BA10 and 27% in BA21 (Figures 11, 12). The most frequent morphological types of PV+/CR+ cells were bipolar and bitufted cells, with round or fusiform somata.

We carried out an additional experiment to analyze the percentage of colocalization of PV+ cells that were also labeled for SCGN. We counted the total number of double-labeled cells through 1,100–1,400 μm-wide columns across the entire thickness of the cortex (from the pial surface to the white matter) in 50 μm-thick sections from both BA10 and BA21. We examined 1,946 PV+ cells in BA10 and 1,329 PV+ cells in BA21 (Supplementary Table S5). Approximately 55% of PV+ cells were also SCGN+ in BA10, whereas in BA21 this percentage was 81%.

Collectively, these results further support the notion that SCGN+ cells include multiple neurochemical subtypes whose abundance varies according to area and cortical layer.

## 4. Discussion

In this study, we provided a detailed description of SCGN+ neurons in the human frontal (BA10) and temporal (BA21) cortices, including the morphological characteristics and the percentage of colocalization of SCGN+ cells with PV, CR and nNOS immunoreactivities, throughout all layers of these two cortical regions. The present study highlights the usefulness of immunocytochemistry for SCGN as a neurochemical marker to distinguish between new subsets of neurons in the human cerebral cortex.

### 4.1. SCGN+/PV+ and SCGN+/CR+ are the most frequent subpopulations of SCGN+ neurons in both BA10 and BA21

The present results provide for the first time a quantitative analysis on the neurochemical profiles of SCGN+ neurons in the human cerebral cortex. We found that the number of SCGN+ is relatively low compared to the total population of cells in both BA10 and BA21, which is in line with the relatively low expression of the SCGN gene in these cortical areas (see gene-specific pages in the Human Protein Atlas www.proteinatlas.org/brain). The pattern of SCGN immunostaining is similar in BA10 and BA21. We found that SCGN+/PV+ and SCGN+/CR+ neurons constitute two major subpopulations of SCGN+. We also found a subpopulation of SCGN+ neurons that have not been described before in human cerebral cortex, namely SCGN+ that are also CR+ and nNOS+ (see below). These neurons represent a small subpopulation of SCGN+ neurons in both BA10 and BA21 (5 and 3%, respectively).

Furthermore, we also found significant regional differences between the frontal and the temporal cortex. For example, although the population of SCGN+/PV+ was similar between BA10 and BA21 approximately 55% of PV+ cells were also SCGN+ in BA10, whereas in BA21 this percentage was higher – approximately 81%. Regarding SCGN+/CR+ neurons, there was a higher proportion of these neurons in BA21 (60%) compared to BA10 (40%). Furthermore, the proportion of SCGN+ that were nNOS+, which represent the smallest population of SCGN+ in both areas, was about three times higher in BA10 than in BA21. These differences between cortical areas are in line with increasing numbers of reports of differences in the pattern of immunostaining for a variety of neurotransmitters, neuropeptides and calcium-binding proteins, as well as in the expression of individual protein-coding genes for transcripts of these substances and transmitter receptor densities for multiple excitatory, inhibitory, ionotropic, and metabotropic receptors, as well as key postsynaptic proteins (DeFelipe, 1993; Inda et al., 2007; Blazquez-Llorca et al., 2010; Lake et al., 2016; Roy et al., 2018; Cadwell et al., 2019; Hodge et al., 2019; Sjöstedt et al., 2020; Yuste et al., 2020; Goulas et al., 2021; Zhong et al., 2022).

### 4.2. Colocalization studies of nNOS with SCGN+/PV+ and SCGN+/CR+

In a previous study performed in the human temporal cortex, we did not observe colocalization of PV or CR with nNOS (Gonzalez-Albo et al., 2001). In the present study, we confirmed that PV does not colocalize with nNOS, but a small number of CR+ also expressed nNOS in layers II, III, and IV, but not in layers I, V or VI. However, comparing between the two studies may present some difficulties due to methodological factors. In González-Albo et al., human tissue was obtained following surgical removal from patients suffering temporal lobe epilepsy (25–30 years old) and the brain tissue was immediately fixed. In the present study, human brain tissue was obtained at autopsy from subjects with no recorded neurological or psychiatric alterations and the post-mortem time between death and brain fixation varied from 1.5 to 4 h. It is possible that some differences were due to the fact that in González-Albo et al. tissue was obtained from epileptic patients, giving rise to the potential for modification of the brain tissue as a result of the clinical characteristics of the (for a recent review, see DeFelipe et al., 2022). Furthermore, post-mortem delay may affect the visualization of nNOS neurons (Young et al., 1992), as well as other neurochemical markers (e.g., Gonzalez-Riano et al., 2017). In addition, the individuals examined in González-Albo et al. were younger (25–30 years old) than those in the present study (45–53 years old), and this difference in age may have an influence on the results. For example, there are age-related changes in the immunostaining for CR and PV in the human cerebral cortex (Bu et al., 2003). Nevertheless, no double-labeling of nNOS with CR or with PV was observed in the monkey cerebral cortex (Smiley et al., 2000), which is in line with the lack of colocalization of NADPH-diaphorase-positive neurons with PV in the monkey cerebral cortex (Yan et al., 1996), since NADPH-diaphorase is colocalized with nNOS (Bredt et al., 1991).

### 4.3. Possible functional significance of the laminar differences between the various subpopulations of SCGN+ in BA10 and BA21

SCGN+ cells were mostly present in layers II, III, and IV in both BA10 and BA21. However, the percentage of the different subpopulation of SCGN+ cells, as determined by the expression of PV, CR and nNOS, was significantly different between layers depending on the subpopulation (Supplementary Tables S1, S2). For example, in layers IV and VI, the subpopulations of SCGN+/PV+ are almost 1.3 and 1.8 times greater in BA21 than in BA10. The subpopulation of SCGN+/CR+ cells is present mostly in layers I, II, and III in both cortical areas, although this is not always the case; e.g., in layers II and IV, the subpopulation of SCGN+/CR+ is, respectively, almost 1.6 and 2 times larger in the BA21. Another example is the subpopulation of SCGN+/nNOS+ cells, which in BA21 are mainly found in layer V, whereas in BA10 they are mainly located in layer I, where most SCGN+ cells colocalize with nNOS. Furthermore, the subpopulation of SCGN+/nNOS+ neurons in layer IV is almost 3.5 times larger in BA10 than in BA21. Thus, our data indicates that the various subpopulations of SCGN cells – as determined by the colocalization with PV, CR, and nNOS – are heterogeneously distributed through the cortical layers.

The functional significance of these findings is difficult to interpret. For example, nNOS is a neurotransmitter that is involved in several functions, including long-term regulation of synaptic transmission; coupling between local cortical blood flow and synaptic activity; neurogenesis; formation of memory; learning; and other cognitive functions (e.g., O’Dell et al., 1991; Schuman and Madison, 1991; Izumi et al., 1992; Estrada and DeFelipe, 1998; Bryan et al., 2009; Zhou and Zhu, 2009; Zoubovsky et al., 2011; Funk and Kwan, 2014). However, multiple brain circuits are involved in these functions and nNOS is expressed in a variety of different subpopulations of neurons based on the presence or absence of neurotransmitters, neuropeptides, or calcium-binding proteins (reviewed in DeFelipe, 1993; DeFelipe, 1997; Caputi et al., 2013; Kubota, 2014; Staiger et al., 2015; Tremblay et al., 2016). Furthermore, the functional significance of nNOS has been examined mostly in experimental animals, but there are species differences in the expression of these neurochemical markers. For example, neurons expressing tyrosine-hydroxylase (TH) are only present in certain species but are particularly abundant in the human cerebral cortex (reviewed in Benavides-Piccione and DeFelipe, 2003). Colocalization studies in the human cerebral cortex revealed that 27% of the moderately labeled nNOS neurons and 3% of the intensely labeled nNOS neurons were also TH+ (Benavides-Piccione and DeFelipe, 2003). Thus, the subpopulation of SCGN+/nNOS+ can be further divided, not only based on the presence or absence of a particular neurochemical marker, but also on the different expression levels of nNOS. All of these features made the interpretation of the possible functional significance of the present results particularly challenging.

### 4.4. Neurons double-immunostained for PV and CR

A large proportion of SCGN+ neurons also express either PV (61% in BA10 and 72% in BA21) or CR (40% in BA10 and 60% in BA21). However, in a previous study performed by Leuba and Saini (1997) in the human visual cortex (BA17 and BA18), it was reported that only 2.5% and 3.3% of PV+ were also CR+ in BA17 and BA18, respectively. Since Leuba and Saini examined different cortical regions, used different immunocytochemical techniques and the characteristics of brain tissue were different to those in the present study (e.g., the post-mortem delay varied between 10 and 20 h and the time of fixation was nearly 1 year in 10% formaldehyde), we assessed this issue in BA10 and BA21. We used triple immunocytochemical techniques to visualize SCGN, PV, and CR in the same sections. However, this immunocytochemical combination did not work for us. Thus, we tried to answer this question indirectly by performing colocalization experiments of PV with CR in both BA10 and BA21. We found that, in BA10, approximately 8% of the PV+ neurons were also CR+; this figure was 27% in the case of BA21. Thus, the extent of overlapping of the subpopulations SCGN+/PV+ and SCGN+/CR+ is lower in BA10 than in BA21.

Finally, in the prefrontal cortex (BA9) of cynomolgus monkeys (Melchitzky et al., 1999) and in the human BA21 (del Rio and DeFelipe, 1996), approximately 25% of CR+ neurons do not express GABA. Since SCGN is expressed in GABAergic neurons and PV represent a major subpopulation of GABAergic cells, we analyzed the percentage of colocalization of PV+ neurons that were also labeled for SCGN. Approximately 55% of PV+ cells were also SCGN+ in BA10, whereas in BA21 this percentage was 81%. Thus, it is possible that CR+ neurons that are SCGN− represent the subpopulation of non-GABAergic CR+ neurons.

In conclusion, the present results further highlight the regional specialization of cortical neurons and underline the importance of performing additional experiments to characterize the subpopulation of SCGN cells in the human cerebral cortex in greater detail.

## Data availability statement

The original contributions presented in the study are included in the article/Supplementary material, further inquiries can be directed to the corresponding author.

## Ethics statement

The studies involving humans were approved by Universidad de Castilla-La Mancha, Albacete (Spain) and Consejo Superior de Investigaciones Científicas (Spain). The studies were conducted in accordance with the local legislation and institutional requirements. The participants provided their written informed consent to participate in this study.

## Author contributions

ST-G: methodology, validation, investigation, and writing. JD: conceptualization, writing – review and editing, supervision, project administration, and funding acquisition. All authors contributed to the article and approved the submitted version.
