# Supplementary material for: Secretagogin as a marker to distinguish between different neuron types in human frontal and temporal cortex
Source: Front Neuroanat. 2023 Nov 1;17:1210502. doi: 10.3389/fnana.2023.1210502 (PMC10646422; doi:10.3389/fnana.2023.1210502)
Supplement: Supplementary file 1 [file Data_Sheet_1.pdf]

*Supplementary Material*

# Secretagogin as a marker to distinguish between different neuron types in human frontal and temporal cortex

**Silvia Tapia-González[1,2,4] and Javier DeFelipe*[1,2,3]**

[1]Laboratorio Cajal de Circuitos Corticales, Centro de Tecnología Biomédica, Universidad Politécnica de Madrid, Pozuelo de Alarcón, 28223 Madrid, Spain

[2]Instituto Cajal, Consejo Superior de Investigaciones Científicas (CSIC), Avda. Doctor Arce 37, 28002 Madrid, Spain

[3]Centro de Investigación Biomédica en Red sobre Enfermedades Neurodegenerativas (CIBERNED), ISCIII, Valderrebollo 5, 28031 Madrid, Spain

[4]Laboratorio de Neurofisiología Celular, Facultad de Medicina, Universidad San Pablo-CEU, CEU Universities, Urb Montepríncipe, 28660 Boadilla del Monte, Madrid, Spain.

*** Correspondence:** Javier DeFelipe defelipe@cajal.csic.es

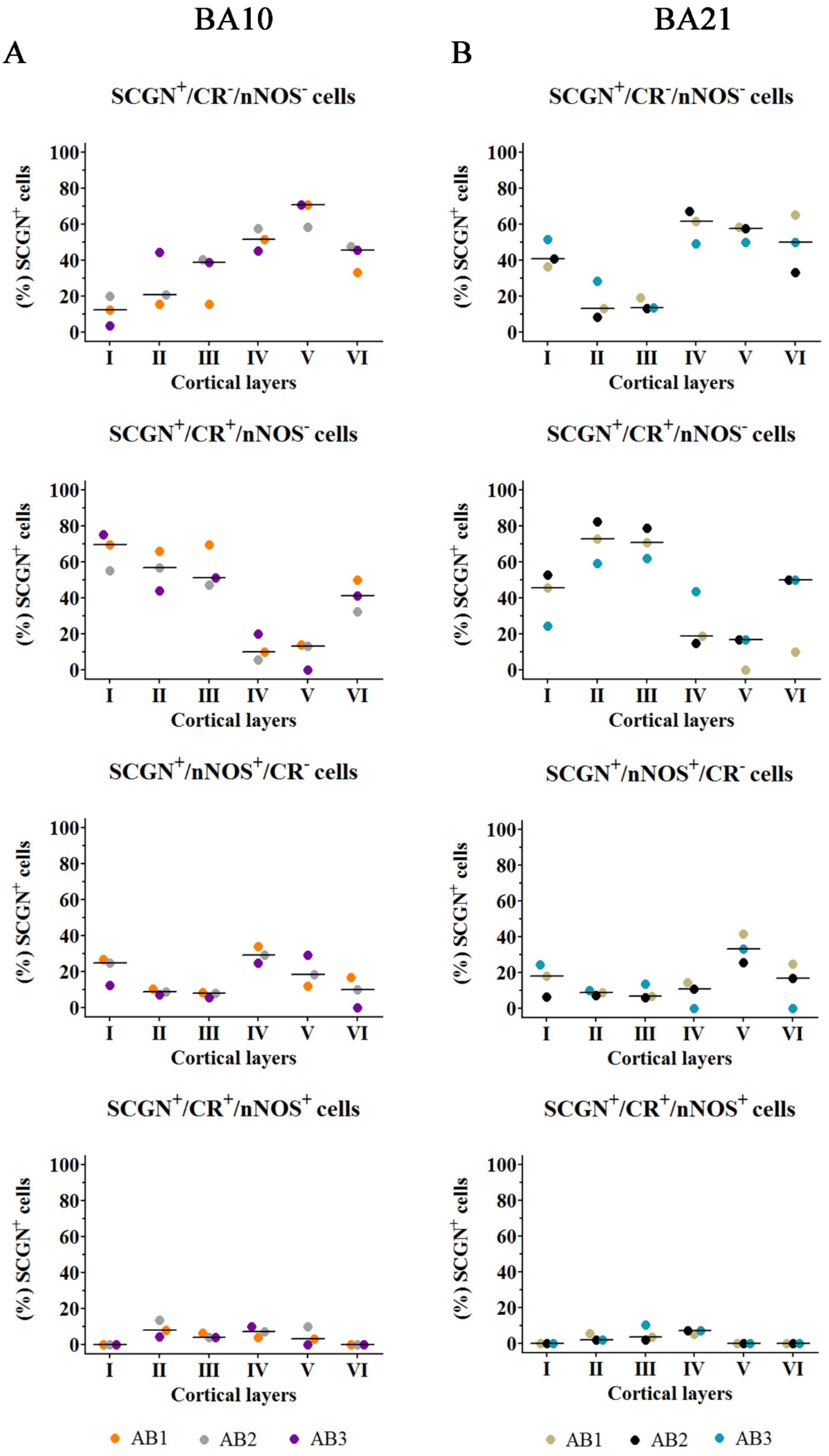


**Supplementary Fig. 1**. Percentages of colocalization of $SCGN^+$ cells with PV and nNOS in all layers per case (AB1, AB2 and AB3) in BA10 (A) and BA21 (B).

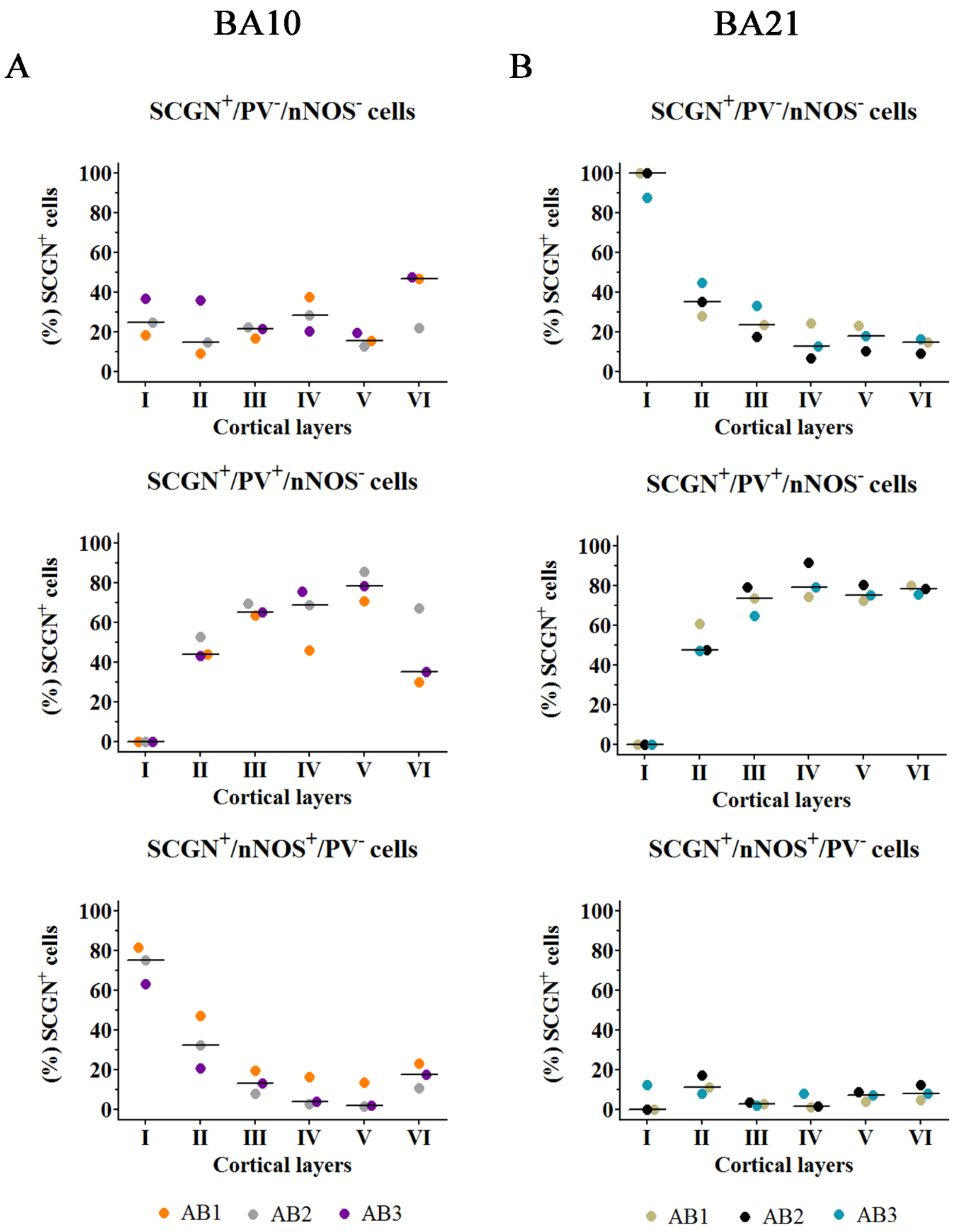


**Supplementary Fig. 2**. Percentages of colocalization of SCGN$^{+}$ cells with CR and nNOS in all layers per case (AB1, AB2 and AB3) in BA10 (A) and BA21 (B).

**Supplementary Table 1.** Statistical comparisons of mean percentages among different subpopulations of SCGN+ cells, whether labeled for PV and nNOS or not, across layers I-VI in both BA10 and BA21. These subpopulations were classified as follows: $SCGN^+/PV^-/nNOS^-$, $SCGN^+/PV^+/nNOS^-$, $SCGN^+/nNOS^+/PV^-$. Significance levels were denoted as follows: *, p < 0.05; **, p < 0.01; ***, p < 0.001. 'ns' indicates no significant differences. 'NA' denotes data not available. In cases marked with a single asterisk (*), the exact p-value has been included**.**

**BA10**

| **$SCGN^+$/** $PV^-/nNOS^-$ | LI | LII | LIII | LIV | LV | LVI |
|---|---|---|---|---|---|---|
| LI | | ns | ns | * 0.023 | ns | ns |
| LII | | | * 0.04 | *** | ns | ** |
| LIII | | | | *** | * 0.033 | ** |
| LIV | | | | | *** | ** |
| LV | | | | | | *** |
| LVI | | | | | | |

| **$SCGN^+$/ $PV^+$/** nNOS- | LI | LII | LIII | LIV | LV | LVI |
|---|---|---|---|---|---|---|
| LI | | ** | *** | *** | *** | *** |
| LII | | | ** | *** | *** | ns |
| LIII | | | | * 0.041 | ** | ** |
| LIV | | | | | ** | *** |
| LV | | | | | | *** |
| LVI | | | | | | |

| **$SCGN^+$/ $nNOS^+$/** $PV^-$ | LI | LII | LIII | LIV | LV | LVI |
|---|---|---|---|---|---|---|
| LI | | * 0.012 | *** | *** | *** | *** |
| LII | | | *** | *** | *** | * 0.017 |
| LIII | | | | *** | *** | * 0.045 |
| LIV | | | | | * 0.048 | *** |
| LV | | | | | | ** |
| LVI | | | | | | |

**BA21**

| **$SCGN^+$/** $PV^-/nNOS^-$ | LI | LII | LIII | LIV | LV | LVI |
|---|---|---|---|---|---|---|
| LI | | *** | *** | *** | *** | *** |
| LII | | | *** | *** | *** | *** |
| LIII | | | | *** | ** | ** |
| LIV | | | | | ** | ** |
| LV | | | | | | ns |
| LVI | | | | | | |

| **$SCGN^+$/ $PV^+$/** $nNOS^-$ | LI | LII | LIII | LIV | LV | LVI |
|---|---|---|---|---|---|---|
| LI | | *** | *** | *** | *** | *** |
| LII | | | *** | *** | *** | *** |
| LIII | | | | *** | ns | * 0.027 |
| LIV | | | | | ** | *** |
| LV | | | | | | ns |
| LVI | | | | | | |

| **$SCGN^+$/ $nNOS^+$/** $PV^-$ | LI | LII | LIII | LIV | LV | LVI |
|---|---|---|---|---|---|---|
| LI | | ** | ns | ** | ns | ns |
| LII | | | *** | *** | ns | ns |
| LIII | | | | ns | ns | ns |
| LIV | | | | | ns | ns |
| LV | | | | | | ns |
| LVI | | | | | | |

**Supplementary Table 2.** Statistical comparisons of mean percentages among different subpopulations of SCGN+ cells, whether labeled for CR and nNOS or not, across layers I-VI in both BA10 and BA21. These subpopulations were classified as follows: $SCGN^+/CR^-/nNOS^-$, $SCGN^+/CR^+/nNOS^-$, $SCGN^+/nNOS^+/CR^-$, $SCGN^+/CR^+/nNOS^+$. Significance levels were denoted as follows: *, $p < 0.05$; **, $p < 0.01$; ***, $p < 0.001$. 'ns' indicates no significant differences. 'NA' denotes data not available. In cases marked with a single asterisk (*), the exact p-value has been included.

**BA10**

| $SCGN^+/CR^-/nNOS^-$ | LI | LII | LIII | LIV | LV | LVI |
|---|---|---|---|---|---|---|
| LI | | * 0.027 | *** | *** | *** | ** |
| LII | | | *** | *** | *** | * 0.03 |
| LIII | | | | *** | *** | ** |
| LIV | | | | | ns | ns |
| LV | | | | | | ns |
| LVI | | | | | | |

| $SCGN^+/CR^+/nNOS^-$ | LI | LII | LIII | LIV | LV | LVI |
|---|---|---|---|---|---|---|
| LI | | ns | * 0.027 | *** | *** | ns |
| LII | | | ** | *** | *** | ns |
| LIII | | | | *** | *** | * 0.02 |
| LIV | | | | | ns | *** |
| LV | | | | | | *** |
| LVI | | | | | | |

| $SCGN^+/nNOS^+/CR^-$ | LI | LII | LIII | LIV | LV | LVI |
|---|---|---|---|---|---|---|
| LI | | * 0.015 | ** | ns | ns | ns |
| LII | | | ns | *** | ns | ns |
| LIII | | | | *** | ns | ns |
| LIV | | | | | ns | ns |
| LV | | | | | | ns |
| LVI | | | | | | |

| $SCGN^+/CR^+/nNOS^+$ | LI | LII | LIII | LIV | LV | LVI |
|---|---|---|---|---|---|---|
| LI | | ns | ns | ns | ns | NA |
| LII | | | ns | * 0.048 | ns | ns |
| LIII | | | | ns | ns | ns |
| LIV | | | | | ns | ns |
| LV | | | | | | ns |
| LVI | | | | | | |

**BA21**

| $SCGN^+/CR^-/nNOS^-$ | LI | LII | LIII | LIV | LV | LVI |
|---|---|---|---|---|---|---|
| LI | | *** | * 0.011 | ns | ns | ns |
| LII | | | ns | *** | *** | *** |
| LIII | | | | *** | *** | *** |
| LIV | | | | | ns | ns |
| LV | | | | | | ** |
| LVI | | | | | | |

| $SCGN^+/CR^+/nNOS^-$ | LI | LII | LIII | LIV | LV | LVI |
|---|---|---|---|---|---|---|
| LI | | *** | *** | ns | * 0.047 | ns |
| LII | | | ns | *** | *** | *** |
| LIII | | | | *** | *** | *** |
| LIV | | | | | ns | ns |
| LV | | | | | | ** |
| LVI | | | | | | |

| $SCGN^+/nNOS^+/CR^-$ | LI | LII | LIII | LIV | LV | LVI |
|---|---|---|---|---|---|---|
| LI | | ns | * 0.037 | ns | ns | ns |
| LII | | | ns | ns | ** | ns |
| LIII | | | | * 0.028 | ** | ns |
| LIV | | | | | ns | ns |
| LV | | | | | | ns |
| LVI | | | | | | |

| $SCGN^+/CR^+/nNOS^+$ | LI | LII | LIII | LIV | LV | LVI |
|---|---|---|---|---|---|---|
| LI | | ns | ns | ns | NA | NA |
| LII | | | * 0.011 | * 0.042 | ns | ns |
| LIII | | | | ** | ns | ns |
| LIV | | | | | NA | ns |
| LV | | | | | | NA |
| LVI | | | | | | |

**Supplementary Table 3.** Statistical comparisons of mean percentages for the subpopulations $SCGN^+/PV^-/nNOS^-$, $SCGN^+/PV^+/nNOS^-$, and $SCGN^+/nNOS^+/PV^-$ across layers I-VI in both BA10 and BA21. Significance levels were denoted as follows: *, $p < 0.05$; **, $p < 0.01$; ***, $p < 0.001$. 'ns' indicates no significant differences. 'NA' denotes data not available. In cases marked with a single asterisk (*), the exact p-value has been included.

| BA10 | | | |
|---|---|---|---|
| **Layers** | **SCGN$^+$**/PV$^-$/nNOS$^-$ | **SCGN$^+$/PV$^+$**/nNOS$^-$ | **SCGN$^+$/nNOS$^+$**/PV$^-$ |
| LI | | | |
| **SCGN$^+$/**PV$^-$/nNOS$^-$ | | *** | *** |
| **SCGN$^+$/PV$^+$**/nNOS$^-$ | | | *** |
| **SCGN$^+$/nNOS$^+$**/PV$^-$ | | | |
| LII | | | |
| **SCGN$^+$/**PV$^-$/nNOS$^-$ | | *** | *** |
| **SCGN$^+$/PV$^+$**/nNOS$^-$ | | | ns |
| **SCGN$^+$/nNOS$^+$**/PV$^-$ | | | |
| LIII | | | |
| **SCGN$^+$**PV$^-$nNOS$^-$ | | *** | * 0.041 |
| **SCGN$^+$PV$^+$**nNOS$^-$ | | | *** |
| **SCGN$^+$ nNOS$^+$**PV$^-$ | | | |
| LIV | | | |
| **SCGN$^+$/**PV$^-$/nNOS$^-$ | | *** | *** |
| **SCGN$^+$/PV$^+$**/nNOS$^-$ | | | *** |
| **SCGN$^+$/nNOS$^+$**/PV$^-$ | | | |
| LV | | | |
| **SCGN$^+$/**PV$^-$/nNOS$^-$ | | *** | ** |
| **SCGN$^+$/PV$^+$**/nNOS$^-$ | | | *** |
| **SCGN$^+$/nNOS$^+$**/PV$^-$ | | | |
| LVI | | | |
| **SCGN$^+$/**PV$^-$/nNOS$^-$ | | * 0.018 | * 0.03 |
| **SCGN$^+$/PV$^+$**/nNOS$^-$ | | | *** |
| **SCGN$^+$/nNOS$^+$**/PV$^-$ | | | |

| **BA21** | | | |
|---|---|---|---|
| **Layers** | **SCGN**$^{+}$/PV$^{-}$/nNOS$^{-}$ | **SCGN**$^{+}$/**PV**$^{+}$/nNOS$^{-}$ | **SCGN**$^{+}$/**nNOS**$^{+}$/PV$^{-}$ |
| LI | | | |
| **SCGN**$^{+}$/PV$^{-}$/nNOS$^{-}$ | | *** | *** |
| **SCGN**$^{+}$/**PV**$^{+}$/nNOS$^{-}$ | | | *** |
| **SCGN**$^{+}$/**nNOS**$^{+}$/PV$^{-}$ | | | |
| LII | | | |
| **SCGN**$^{+}$/PV$^{-}$/nNOS$^{-}$ | | ** | *** |
| **SCGN**$^{+}$/**PV**$^{+}$/nNOS$^{-}$ | | | *** |
| **SCGN**$^{+}$/**nNOS**$^{+}$/PV$^{-}$ | | | |
| LIII | | | |
| **SCGN**$^{+}$/PV$^{-}$/nNOS$^{-}$ | | *** | *** |
| **SCGN**$^{+}$/**PV**$^{+}$/nNOS$^{-}$ | | | *** |
| **SCGN**$^{+}$/**nNOS**$^{+}$/PV$^{-}$ | | | |
| LIV | | | |
| **SCGN**$^{+}$/PV/$^{-}$nNOS$^{-}$ | | *** | *** |
| **SCGN**$^{+}$/**PV**$^{+}$/nNOS$^{-}$ | | | *** |
| **SCGN**$^{+}$/**nNOS**$^{+}$/PV$^{-}$ | | | |
| LV | | | |
| **SCGN**$^{+}$/PV$^{-}$/nNOS$^{-}$ | | *** | *** |
| **SCGN**$^{+}$/**PV**$^{+}$/nNOS$^{-}$ | | | *** |
| **SCGN**$^{+}$/**nNOS**$^{+}$/PV$^{-}$ | | | |
| LVI | | | |
| **SCGN**$^{+}$/PV$^{-}$/nNOS$^{-}$ | | *** | *** |
| **SCGN**$^{+}$/**PV**$^{+}$/nNOS$^{-}$ | | | *** |
| **SCGN**$^{+}$/**nNOS**$^{+}$/PV$^{-}$ | | | |

**Supplementary Table 4.** Statistical comparisons of mean percentages for the subpopulations $SCGN^+/CR^-/nNOS^-$, $SCGN^+/CR^+/nNOS^-$, $SCGN^+/nNOS^+/CR^-$, and $SCGN^+/CR^+/nNOS^+$ across layers I-VI in both BA10 and BA21. Significance levels were denoted as follows: *, $p < 0.05$; **, $p < 0.01$; ***, $p < 0.001$. 'ns' indicates no significant differences. 'NA' denotes data not available. In cases marked with a single asterisk (*), the exact p-value has been included.

| BA10 | | | | |
|---|---|---|---|---|
| **Layers** | **$SCGN^+$**/$CR^-$/$nNOS^-$ | **$SCGN^+$/$CR^+$**/$nNOS^-$ | **$SCGN^+$/$nNOS^+$**/$CR^-$ | **$SCGN^+$/$CR^+$/$nNOS+$** |
| LI | | | | |
| **$SCGN^+$**/$CR^-$/$nNOS^-$ | | ** | ns | *** |
| **$SCGN^+$/$CR^+$**/$nNOS^-$ | | | ns | *** |
| **$SCGN^+$/$nNOS^+$**/$CR^-$ | | | | *** |
| **$SCGN^+$/$CR^+$/$nNOS^+$** | | | | |
| LII | | | | |
| **$SCGN^+$**/$CR^-$/$nNOS^-$ | | *** | *** | *** |
| **$SCGN^+$/$CR^+$**/$nNOS^-$ | | | *** | *** |
| **$SCGN^+$/$nNOS^+$**/$CR^-$ | | | | ns |
| **$SCGN^+$/$CR^+$/$nNOS^+$** | | | | |
| LIII | | | | |
| **$SCGN^+$**/$CR^-$/$nNOS^-$ | | *** | *** | *** |
| **$SCGN^+$/$CR^+$**/$nNOS^-$ | | | *** | *** |
| **$SCGN^+$/$nNOS^+$**/$CR^-$ | | | | ns |
| **$SCGN^+$/$CR^+$/$nNOS^+$** | | | | |
| LIV | | | | |
| **$SCGN^+$**/$CR^-$/$nNOS^-$ | | *** | *** | *** |
| **$SCGN^+$/$CR^+$**/$nNOS^-$ | | | *** | ns |
| **$SCGN^+$/$nNOS^+$**/$CR^-$ | | | | *** |
| **$SCGN^+$/$CR^+$/$nNOS^+$** | | | | |
| LV | | | | |
| **$SCGN^+$**/$CR^-$/$nNOS^-$ | | *** | *** | *** |
| **$SCGN^+$/$CR^+$**/$nNOS^-$ | | | ns | ns |
| **$SCGN^+$/$nNOS^+$**/$CR^-$ | | | | ns |
| **$SCGN^+$/$CR^+$/$nNOS^+$** | | | | |
| LVI | | | | |
| **$SCGN^+$**/$CR^-$/$nNOS^-$ | | ns | * 0.043 | *** |
| **$SCGN^+$/$CR^+$**/$nNOS^-$ | | | * 0.049 | *** |
| **$SCGN^+$/$nNOS^+$**/$CR^-$ | | | | *** |
| **$SCGN^+$/$CR^+$/$nNOS^+$** | | | | |

| BA21 | | | | |
|---|---|---|---|---|
| **Layers** | **SCGN$^{+}$**/CR$^{-}$/nNOS$^{-}$ | **SCGN$^{+}$/CR$^{+}$**/nNOS$^{-}$ | **SCGN$^{+}$/nNOS$^{+}$**/CR$^{-}$ | **SCGN$^{+}$/CR$^{+}$/nNOS$^{+}$** |
| LI | | | | |
| **SCGN$^{+}$**/CR$^{-}$/nNOS$^{-}$ | | ns | ns | *** |
| **SCGN$^{+}$/CR$^{+}$**/nNOS$^{-}$ | | | ns | *** |
| **SCGN$^{+}$/nNOS$^{+}$**/CR$^{-}$ | | | | *** |
| **SCGN$^{+}$/CR$^{+}$/nNOS$^{+}$** | | | | |
| LII | | | | |
| **SCGN$^{+}$**/CR$^{-}$/nNOS$^{-}$ | | *** | ** | *** |
| **SCGN$^{+}$/CR$^{+}$**/nNOS$^{-}$ | | | *** | *** |
| **SCGN$^{+}$/nNOS$^{+}$**/CR$^{-}$ | | | | ns |
| **SCGN$^{+}$/CR$^{+}$/nNOS$^{+}$** | | | | |
| LIII | | | | |
| **SCGN$^{+}$**/CR$^{-}$/nNOS$^{-}$ | | *** | *** | *** |
| **SCGN$^{+}$/CR$^{+}$**/nNOS$^{-}$ | | | *** | *** |
| **SCGN$^{+}$/nNOS$^{+}$**/CR$^{-}$ | | | | ** |
| **SCGN$^{+}$/CR$^{+}$/nNOS$^{+}$** | | | | |
| LIV | | | | |
| **SCGN$^{+}$**/CR$^{-}$/nNOS$^{-}$ | | *** | *** | *** |
| **SCGN$^{+}$/CR$^{+}$**/nNOS$^{-}$ | | | ns | ** |
| **SCGN$^{+}$/nNOS$^{+}$**/CR$^{-}$ | | | | ns |
| **SCGN$^{+}$/CR$^{+}$/nNOS$^{+}$** | | | | |
| LV | | | | |
| **SCGN$^{+}$**/CR$^{-}$/nNOS$^{-}$ | | ** | ns | *** |
| **SCGN$^{+}$/CR$^{+}$**/nNOS$^{-}$ | | | *** | *** |
| **SCGN$^{+}$/nNOS$^{+}$**/CR$^{-}$ | | | | *** |
| **SCGN$^{+}$/CR$^{+}$/nNOS$^{+}$** | | | | |
| LVI | | | | |
| **SCGN$^{+}$**/CR$^{-}$/nNOS$^{-}$ | | ns | ** | *** |
| **SCGN$^{+}$/CR$^{+}$**/nNOS$^{-}$ | | | ** | *** |
| **SCGN$^{+}$/nNOS$^{+}$**/CR$^{-}$ | | | | *** |
| **SCGN$^{+}$/CR$^{+}$/nNOS$^{+}$** | | | | |

**Supplementary Table 5.** Number of $PV^+$ cells examined and the percentage (mean±SEM) of $PV^+$ neurons that are either $SCGN^+$ or $SCGN^-$ in all layers of BA10 and BA21.

| **Nº $PV^+$ cells** (all cases) | | **Nº $PV^+$ cells/area** (all cases) | | | **% $PV^+$ cells /case/area** | | | **% $PV^+$ cells** (all cases) | |
|---|---|---|---|---|---|---|---|---|---|
| **BA10** | **BA21** | | **BA10** | **BA21** | **Case** | **BA10** | **BA21** | **BA10** | **BA21** |
| 1946 | 1329 | **$PV^+/SCGN^+$** | 956 | 1065 | AB1 | 47.20±2.80 | 75.99±0.05 | 54.87±3.03 | 81.32±1.68 |
| | | | | | AB2 | 59.66±1.20 | 82.61±1.76 | | |
| | | | | | AB3 | 57.76±5.05 | 81.64±2.63 | | |
| | | **$PV^+/SCGN^-$** | 782 | 264 | AB1 | 52.80±2.80 | 24.01±0.05 | 45.13±3.03 | 18.68±1.68 |
| | | | | | AB2 | 40.34±1.20 | 17.39±1.76 | | |
| | | | | | AB3 | 42.24±5.05 | 18.36±2.63 | | |
